# pISTil: a pipeline for yeast two-hybrid Interaction Sequence Tags identification and analysis

**DOI:** 10.1186/1756-0500-2-220

**Published:** 2009-10-29

**Authors:** Johann Pellet, Laurène Meyniel, Pierre-Olivier Vidalain, Benoît de Chassey, Lionel Tafforeau, Vincent Lotteau, Chantal Rabourdin-Combe, Vincent Navratil

**Affiliations:** 1 U851, INSERM, 21 avenue Tony Garnier, F-69007, Lyon, France; 2 Université de Lyon, Lyon, France; 3 IFR128, Université Lyon 1, Lyon, France; 4 Laboratoire de Génomique Virale et Vaccination, Institut Pasteur, CNRS, URA 3015, Paris, France; 5 UMR754, INRA, ENVL, Lyon, France; 6 Current address: Centre de RMN à Très Hauts Champs, CNRS, 5 rue de la Doua, 69100, Villeurbanne, France

## Abstract

**Background:**

High-throughput screening of protein-protein interactions opens new systems biology perspectives for the comprehensive understanding of cell physiology in normal and pathological conditions. In this context, yeast two-hybrid system appears as a promising approach to efficiently reconstruct protein interaction networks at the proteome-wide scale. This protein interaction screening method generates a large amount of raw sequence data, *i.e*. the ISTs (Interaction Sequence Tags), which urgently need appropriate tools for their systematic and standardised analysis.

**Findings:**

We develop pISTil, a bioinformatics pipeline combined with a user-friendly web-interface: (*i*) to establish a standardised system to analyse and to annotate ISTs generated by two-hybrid technologies with high performance and flexibility and (*ii*) to provide high-quality protein-protein interaction datasets for systems-level approach. This pipeline has been validated on a large dataset comprising more than 11.000 ISTs. As a case study, a detailed analysis of ISTs obtained from yeast two-hybrid screens of Hepatitis C Virus proteins against human cDNA libraries is also provided.

**Conclusion:**

We have developed pISTil, an open source pipeline made of a collection of several applications governed by a Perl script. The pISTil pipeline is intended to laboratories, with IT-expertise in system administration, scripting and database management, willing to automatically process large amount of ISTs data for accurate reconstruction of protein interaction networks in a systems biology perspective. pISTil is publicly available for download at .

## Findings

Systems biology focuses, in part, on exhaustive and accurate reconstruction of molecular interaction networks, which support cellular machinery, *i.e *interactomes, under physiological or pathological conditions.

Molecular interactions data related to human and model organisms are currently being integrated in generalist databases, such as INTACT [1], MINT [2] or STRING [3]. Some other databases are more specialised, as for instance VirHostNet, a knowledgebase devoted to virus-host interactions that allows analysis and visualisation of infection at the systems level [4]. One of the main sources of protein-protein interactions deposited in these public databases is generated by yeast two-hybrid (Y2H) technology. Indeed, Y2H allows high-throughput screening of direct physical protein-protein interactions at a proteome scale, but requires the sequencing of hundreds to thousands of cellular preys per experiment. These prey sequences extracted from yeast positive colonies are referred to ISTs, *i.e*. Interaction Sequence Tags [5]. Dedicated tools were developed to deal with high-throughput sequencing of ESTs (Expressed Sequence Tags) in transcriptome-based studies of cell lines, tissues or whole organ libraries in different physiological contexts and mainly rely on Phred functionalities [6]. However, these tools are not fully adapted for ISTs analysis. For instance, information related to cDNA libraries vectors has to be used to unambiguously define the IST reading "frame" and to eliminate cDNA inserts that have been cloned into abnormal reading frame or correspond to untranslated mRNA regions (UTRs). The reconstruction of a high-quality protein interactome dramatically depends on this unambiguous annotation of ISTs.

In this paper, we present pISTil, a fully automated pipeline combined to a web-based interface, which are specifically devoted to ISTs identification and analysis. The pISTil system is highly flexible and allows: (*i*) systematic and fast assignment of ISTs to a unique protein accession number; (*ii*) annotation of "in frame" ISTs (*i.e*., ISTs with prey cDNA inserts are in frame with Gal4 transactivation domain) or "not in frame" ISTs (*i.e*., ISTs with prey cDNA inserts are not in frame with Gal4 transactivation domain); (*iii*) sequence quality filtering, manual checking and visualisation of annotated ISTs through a user-friendly web interface and (*iv*) export of protein-protein interactions in multiple formats, such as MIMIx standard format [7]. The pISTil annotation procedure has been tested and validated with more than 11.000 ISTs generated by Y2H screening of human cDNA libraries. This comprehensive analysis led us to define optimal thresholds that reduced the noise to signal ratio associated to ISTs. As a case study, the pISTil pipeline and its web interface utility were illustrated through the analysis of Y2H screens that have been successfully used to reconstruct a relevant HCV-human protein infection network [8].

## Implementation

**Figure 1:**
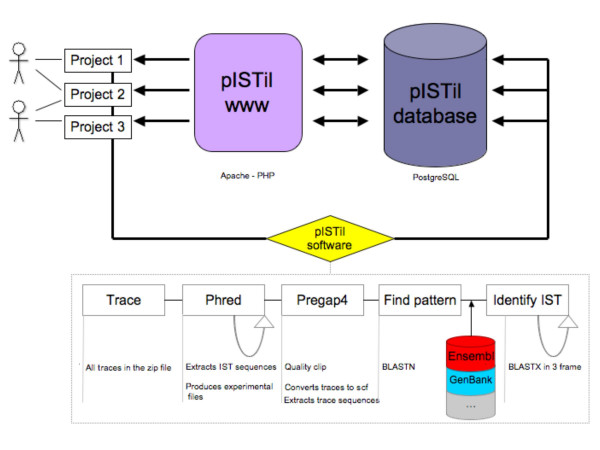
**pISTil pipeline workflow**. pISTil is organised around three major components. The pISTil software analyses chromatogram files (traces) that are organised by project. In this figure, three different projects are shown with two users. The pISTil web application provides a web interface for the visualization of projects results. The pISTil database integrates all IST analysis meta-information.

The pISTil pipeline is implemented using a collection of open source program and bioinformatics tools such as Perl, Bioperl, Staden, PHP, Java, NCBI-Blast Toolkit and the PostgreSQL database system (Figure 1). Information on installing and running pISTil is given in the documentation distributed with pISTil [see Additional file 1].

First of all, IST chromatogram files - in ABI (Applied Biosystems INC) or SCF (Standard Chromatogram Format) formats - are filtered by Phred-pregap4 software [9,10] in order to extract nucleic sequences and their associated quality value. The resulting nucleic sequence of each IST is then translated into three frames and aligned against a protein sequence database (as defined in the configuration file by the users) by using BLASTX alignment software [11,12]. Only alignment information for the best hit is subsequently retained. In addition, identification of Gal4 transactivation domain (Gal4-AD) on ISTs allows the true delineation of "in frame" and "not in frame" ISTs [5] that may lead to false positive protein-protein interaction annotation. Even though translational frame-shift is possible in yeast, "not in frame" ISTs may be more prone to errors related to the irrelevant nature of associated proteins. All information generated by the IST pipeline are stored into the pISTil database, such as sequence quality of ISTs, identity percentage of ISTs, E-value, alignment position, the reading frame and protein sequence database source (Ensembl, RefSeq, etc.). Other meta-data supplied by users, such as origin (host organism, tissue origin, cell type), bait protein accession number/name that was used for the Y2H screen (GenBank accession number) and description of cDNA libraries constructions that have been used for the Y2H screens, are also integrated into the pISTil database.

## pISTil pipeline validation

An experimental dataset of 11.658 ISTs obtained from more than 300 Y2H screens was tested in order to validate the pISTil pipeline (unpublished; data not shown).

**Figure 2:**
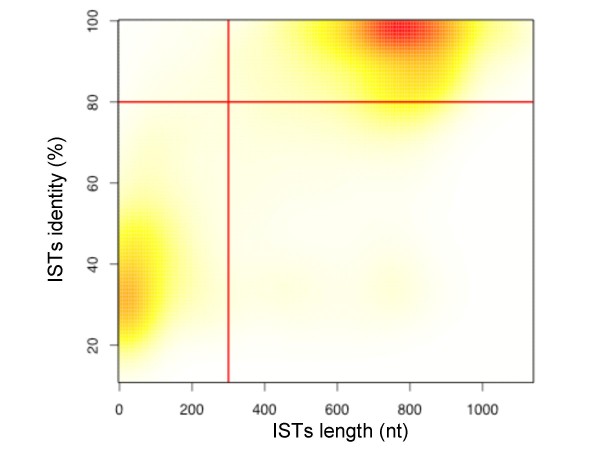
**pISTil pipeline validation**. Correlation between the percentage of identity of whole ISTs with RefSeq protein sequences and the length of these ISTs filtered with a Phred score above 13 (n = 11.658 ISTs). Red-yellow colours gradient is correlated to the density of points (red and yellow correspond respectively to high and low density regions).

We statistically assessed the stringency of our filter parameters to define optimal thresholds that maximise the true positive rate associated to virus-host protein-protein interactions. One major drawback related to high-throughput sequencing of ISTs is the generation of sequences of poor quality [5]. Indeed, PCR-based procedures used to extract prey cDNA directly from yeast colonies are not optimal in term of yield and specificity, and often generate poor quality templates for sequencing reactions [13]. Because high quality sequences retrieved from Y2H screens are often short (*i.e*. <300 bp), filtering ISTs based only on sequence length appears in this context inadequate. In Figure 2, correlation between the length of ISTs filtered with a Phred score > 13 (probability of incorrect base call < 5/100) and the percentage of identity between ISTs and annotated proteins (RefSeq) shows that ISTs with long stretch of high-quality nucleotides are correctly discriminated at the 80 percent of identity threshold. Thus, only filtering on identity may be appropriate to recover those short quality sequences that are discarded by applying additional Phred quality cut-off.

In this study, ISTs were thus considered as highly significant if they follow these criteria: (*i*) an identity threshold superior or equal to 80% with an e-value threshold inferior or equal to 10e^-10 ^and (*ii*) a protein product defined as "in frame".

## HCV-human protein-protein interactions analysis, a case study

pISTil was previously applied to analyse ISTs generated from Y2H screens [8]. In this study, 27 constructs encoding full-length HCV mature proteins or discrete domains were used as baits to screen human cDNA prey libraries. As a case study, 1.158 chromatogram files related to two of these screens were processed using the "ist_analyse.pl" program.

Analysis showed that 50% of the sequences passed through the first filters (identity ≥ 80%, e-value ≤ 1e^-10^). As described above, this low retention rate is commonly observed when extracting ISTs directly from yeast by PCR. This success rate dramatically increased when rescuing DNA templates extracted from yeast by bacteria transformation, but this later procedure was much more time consuming (data not shown). Even if 77% of ISTs passing through the first filter are "in frame", the remaining 23% ISTs are "not in frame" and might be considered as "true positives" because of translation mechanisms existing in yeast that allow stop-codon reading through and frame-shift correction for a significant fraction of the preys [14-16]. Altogether, our stringent ISTs identification pipeline leads to characterise unambiguously roughly 40% of the sequences (443/1158) and defined for 10 viral proteins used as baits 132 distinct protein interactions and 117 unique host protein partners. Based on these criteria, these protein-protein interactions were alternatively confirmed by GST pull-down validation with a success rate of 80% [8], underlying the efficiency of the pISTil pipeline.

Additional thresholds might be used to reduce the false positive rate of ISTs assignation, for instance the number of independent ISTs observations for each non-redundant interaction. Indeed previous studies have shown that protein-protein interactions defined by more than three ISTs exhibit a high rate of confirmation with alternative interaction detection methods such as co-immunoprecipitation or pull-down [5].

## pISTil Web interface and utility

In order to manually check all information associated to ISTs annotation, a web interface was designed.

**Figure 3:**
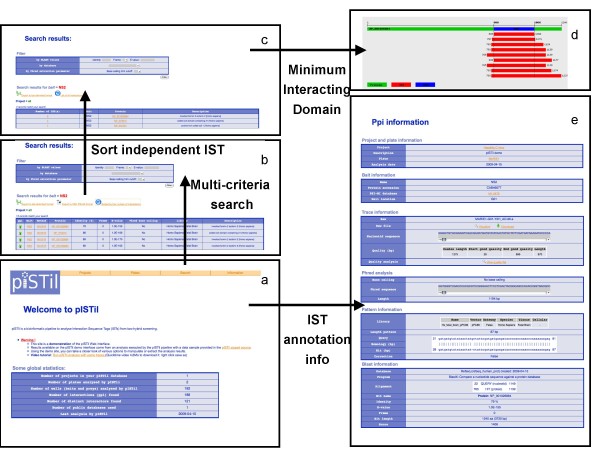
**pISTil web interface screenshots**. Complete details on how to use the interface is given in the pISTil documentation [see Additional file 1]. **a **- Home page: . **b **- Multicriteria search result page: . **c **- Search independent IST result page: . **d - **Interaction domain result page - . **e **- PPI information result page - .

The pISTil web interface was fully implemented in PHP/PostgreSQL [see Additional file 1]. A demonstration of the web site capabilities is available at . Throughout this web interface, users can easily access yeast two-hybrid meta-data, such as project, bait, plate and ISTs information (see Figure 3 and documentation). An advanced search interface allows querying and ranking protein-protein interaction annotations using multiple-criteria, such as quality of ISTs, "in frame" ISTs annotation, percentage of identity of ISTs, e-value and the number of independent ISTs observations associated with non redundant protein-protein interactions. The results of interactions between proteins associated with baits and preys can be displayed as a HTML table. This table can be sorted according to the number of independent ISTs associated with non-redundant protein-protein interactions (Figure 3). Functionality related to the design and the visualisation of the minimal interacting domain is also provided for further experimental validation. This minimal interacting domain is obtained by extracting the minimal common protein sequence from multiple alignment of independent IST defining a non-redundant protein-protein interaction (Figure 3).

By using compliant PSI-MI (Proteomics Standards Initiative-Molecular Interaction) standard as file format for molecular interaction output and by carefully following MIMIx guidelines, efforts have also been made to facilitate unified exchange of protein-protein interaction data with the main public interaction data providers, such as those belonging to the IMEx consortium.

## Conclusion

We have developed pISTil, a pipeline for large-scale identification and analysis of ISTs data generated by yeast two-hybrid approach. This application is dedicated to laboratories willing to automatically process, easily visualise and efficiently share yeast two-hybrid data. The use of such a standard approach will facilitate comparisons of datasets and will improve quality of protein-protein interaction network reconstruction in systems biology projects. Finally, next generation sequence tags project relying on cDNA libraries may also take advantage of this open source and efficient pipeline. pISTil is available under the GNU General Public License and may be downloaded from its project website.

## Availability and requirements

• **Project name**: pISTil

• **Project home page**: 

• **Operating system(s)**: Running on Mac OS × 10.4× or higher, Linux (Linux 2.6.18-1.2798.fc6) and Unix Solaris systems (SunOS 5.10)

• **Programming language**: Perl 5.0 or higher, PHP (php4 or php5), PostgreSQL 8. × or higher

• **Other requirements**: Phred, Apache 2.0, Staden 1.6.0, NCBI BLAST Toolkit

• **License**: GNU General Public License

• **Any restrictions to use by non-academics**: License require

## Abbreviations

bp: base pairs; EST: Expressed Sequence Tag; IST: Interaction Sequence Tag; nt: nucleotide; PSI-MI: Proteomics Standards Initiative-Molecular Interaction; Y2H: Yeast Two-Hybrid; ABI: Applied Biosystems INC; SCF: Standard Chromatogram Format; HTML: Hypertext Markup Language.

## Competing interests

The authors declare that they have no competing interests.

## Authors' contributions

VN designed the study and drafted the manuscript. JP developed the pISTil system, the pISTil web interface and wrote the pISTil documentation. LM contributed to the development of the pISTil web interface, the PSI-MI XML export; tested the pISTil software, corrected the pISTil documentation and manuscript. BdC, LT, POV contributed to the design of pISTil algorithm and corrected the manuscript. VL and CRC provided funding and corrected the manuscript. All authors read and approved the final manuscript.

## Supplementary Material

Additional file 1 **The pISTil documentation**. This documentation gives detailed information on how to install, run and use the pipeline as well as its associated web interface. Click here for file
