# Supplementary material for: pISTil: a pipeline for yeast two-hybrid Interaction Sequence Tags identification and analysis
Source: BMC Res Notes. 2009 Oct 29;2:220. doi: 10.1186/1756-0500-2-220 (PMC2776022; doi:10.1186/1756-0500-2-220)
Supplement: Additional file 1 — The pISTil documentation. This documentation gives detailed information on how to install, run and use the pipeline as well as its associated web interface. [file 1756-0500-2-220-S1.pdf]

p I S T i l

a pipeline for Interaction Sequence Tags identification and analysis

Release v1.0.6: 2/10/2009
Johann PELLET

pISTil documentation

Table of contents:

ABOUT THIS DOCUMENTATION 5

ABOUT THE LICENCE AGREEMENT 5

I. INTRODUCTION 6

II. REQUIREMENTS 6

1. PostgreSQL -- http://www.postgresql.org 7
2. Apache Web Server -- http://www.apache.org 7
3. MAMP (for Macintosh OS system) -- http://www.mamp.info 7
4. Perl -- http://www.cpan.org 7
5. Standard Perl modules -- http://www.cpan.org 7
6. Bioperl version 1.5.2 or higher -- http://www.bioperl.org 7
7. NCBI BLAST Toolkit -- ftp://ftp.ncbi.nih.gov/blast/executables/release/ 7
8. Staden package -- http://staden.sourceforge.net 8
9. Phred software - http://www.phrap.org/phredphrapconsed.html 8
10. JDK -- http://www.sun.com 8
11. csh shell 8

III. INSTALLATION AND CONFIGURATION 9

1. Downloading and unzipping pISTil 9
2. Creating the pISTil database: 9
3. Setting up the pISTil configuration file 10
4. Downloading and creating the BLAST databases: 12
5. Creating the pattern BLAST database 12
6. Configuring "the bait parameter file" 12
7. Setting up the pISTil interface 13
8. Edit library and vector data 14

IV. RUNNING pISTil: 15

1. Quick start: 15
2. Running with your own bait parameter file: 15
3. Example with the two HCV datasets: 15
4. Miscellaneous 20
5. pISTil processing time 20

V. pISTil WEB INTERFACE 21

1. Viewing projects 21
2. Viewing plates 22
3. Viewing protein-protein interaction (ppi) 25
4. Search page 27
5. PSI-MI export 30
6. Information 32

VI. pISTil PROGRAM FLOW 34

VII. pISTil FILES: 37

VIII. BUGS AND PROBLEMS 39

1. Environment variable STADLIB 39
2. Stash not found 39

Annex 40

Annex 1: pISTil Entity-relationship (ER) diagram 40
Annex 2: PostgreSQL table listing 42
Annex 3: Config_analyse.pm example, working with both HCV datasets 45
Annex 4: Define_bait examples 46
Annex 5: config.inc example, working with both HCV datasets 47
Annex 6: PSI-MI from Ontology browser for sequence databases 48
Annex 7: Vector pPC86 used for the HCV examples and its pattern 49
Annex 8: pISTil workflow schema 50
Annex 9: The trace sequence, according to several Phred processing options 51
Annex 10: Interaction Sequence Tag (IST) identification pipeline statistics 52
Annex 11: Schema of the BLASTN and the split correction to find the cDNA start. 53

ABOUT THIS DOCUMENTATION

This documentation is intended to inform informatics or bioinformatics users on how to use **pISTil**. Several formatting conventions are used throughout this documentation:

> *Commands are written in this style.*
>
> pISTil output is written in this style.
>
> **Names of programs, packages are written in this style.**
>
> References to web sites are written in this style.

ABOUT THE LICENCE AGREEMENT

pISTil v1.0.6 29/07/2009
INSERM U851, I-MAP team,
21 Avenue Tony Garnier, Lyon F-69007 France

Copyright (C) 2009 I-MAP INSERM U851

All scripts, programs and applications used are free software; you can redistribute them and/or modify them under the terms of the GNU General Public License as published by the Free Software Foundation; either version 2 of the License, or (at your option) any later version.

They are distributed in the hope that it will be useful, but WITHOUT ANY WARRANTY; without even the implied warranty of MERCHANTABILITY or FITNESS FOR A PARTICULAR PURPOSE. See the GNU General Public License for more details.

You should have received a copy of the GNU General Public License along with pISTil; if not, write to the Free Software Foundation, Inc., 59 Temple Place, Suite 330, Boston, MA 02111-1307 USA

I. INTRODUCTION

pISTil (a **p**ipeline for **I**nteraction **S**equence **T**ag **i**dentification and ana**l**ysis) is a collection of scripts and programs - running on both Linux and MacOS X systems - for fast analysis of large yeast two-hybrid sequence datasets. **pISTil** is composed of (i) a database, (ii) a web interface and (iii) a **perl** script.

The **pISTil perl** script takes as input files sequence chromatogram data generated from automated sequencing technology, in either (i) Applied Biosystems INC. (ABI) format or (ii) Standard Chromatogram Format (SCF).

The **pISTil** package provides a combination of functionalities that allow:

- to convert trace files to bases and quality indices by using **Phred** software
- to analyse chromatograms with different **Phred** parameters and/or **BlastX** protein sequence databases
- to automatically carry out sequence alignments and store aligned sequences
- to store results from all analysis in a relational database
- to apply different search criteria, such as the frequency of interaction, the number of distinct interactors etc… and different filters (E-value, identity, frame)
- to export lists of interaction in different file formats (Excel, PSI-MI: **P**roteomics **S**tandards **I**nitiative - **M**olecular **I**nteractions)

The **pISTil** distribution includes, as a case study, the HCV (**H**epatitis **C** **V**irus) dataset produced by the IMAP team (**I**nfection **MAP**ping) that you can be used with the tutorial described in section IV.3.

Note: **pISTil** was developed to analyse large datasets of cDNA sequences produced by high-throughput yeast two hybrid screens. However, it can be extended to other applications dedicated to protein-protein interaction identification, like MAPPIT (MAmmalian Protein-Protein Interaction Trap), LUMIER (luminescence-based mammalian interactome mapping) or PCA (protein complementation assay) by modifying the open source code available at http://sourceforge.net/projects/pistil .

II. REQUIREMENTS

We have tested the software on MacOS X 10.5.X and Linux, and would recommend the following system specifications:

- Operating Systems:
  - Mac OS X 10.4.x or higher.
  - Linux Fedora 2.6.18-1.2798.fc6 or equivalent
- Server Specifications:
  - 1.5 GB of hard drive space
  - 1 GB of RAM or better

pISTil is distributed as a source code for Linux and Macintosh OS X systems.
It runs on top of several software packages. These must be installed and configured before you can run **pISTil**.
You can access to this requirements list on this page:

1. **PostgreSQL -- http://www.postgresql.org**

PostgreSQL is a powerful, open source relational database system to store various pieces of information: sequences, annotation, alignments, etc. A relational database is an ideal way to store large datasets as it allows very fast storing and retrieval information. To run **pISTil**, you must be able to create and access a **PostgreSQL** database. A diagram of the **pISTil** database structure is included at the end of this document (See Annex 1).

2. **Apache Web Server -- http://www.apache.org**

The **Apache** web server is the industry standard open source web server for Unix and Windows systems. For Macintosh OS system, **MAMP** can be used.

3. **MAMP (for Macintosh OS system) -- http://www.mamp.info**

MAMP installs easily **Apache**, **PHP** and **Mysql** for Mac OS X users.

4. **Perl -- http://www.cpan.org**

Perl is a high-level programming language and **CPAN** is the **C**omprehensive **P**erl **A**rchive **N**etwork, a large collection of **Perl** software and documentation.

The **Perl** interpreter is usually present on most Unix distributions. Type *perl -v* at the command line to find which version of **Perl** is available on your system (version 5.8.8 or higher is preferred).

Note: If **Perl** is not installed under /usr/bin/perl, either make a soft link at the location where **Perl** is installed. Alternatively, you can modify the first line of all **Perl** scripts in the **pISTil** directory so that they point to the correct location.

5. **Standard Perl modules -- http://www.cpan.org**

The following **Perl** modules can be found on the CPAN and must be installed for **pISTil** to work:

- **CGI**
- **DBI**
- **Carp**
- **Text::Wrap**
- **Math::BigFloat**

6. **Bioperl version 1.5.2 or higher -- http://www.bioperl.org**

BioPerl is a collection of **Perl** modules devoted to bioinformatics. It is not usually installed on Unix systems and has to be installed separately. You can find out if it is installed by running *perl –MBIO::Perl –e '1'* from a terminal window. If it doesn't return an error, then **BioPerl** is installed.

7. **NCBI BLAST Toolkit -- ftp://ftp.ncbi.nih.gov/blast/executables/release/**

BLAST (**B**asic **L**ocal **A**lignment **S**earch **T**ool) is used to search in a formatted database for sequences that show similarities to a query sequence. Within **pISTil**, it is used to identify sequences that show significant similarities to a well-annotated protein, and thereby to putatively assign protein accession number to each IST (**I**nteraction **S**equence **T**ag). Two binaries are required, **blastall** (which carries out the search) and **formatdb** (which prepares a database for searching).

8. Staden package -- http://staden.sourceforge.net

pISTil uses **Pregap4**, a **Staden package** program, to prepare sequence chromatogram data for analysis. **pISTil** has been tested with rel-1-6-0 release of **Staden package**. Install the package as described in the accompanying documentation. Make sure:

- to include the directory where the **Staden** binaries reside in your path.
- to set the STADENROOT environment variable.
- to source the appropriate **Staden** script as described in the **Staden** documentation.

For **pISTil**, you have to set the 'STADLIB' environment variable. If you use **sh**, or variants such as **bash**, and install **Staden** package in /usr/local/staden , set 'STADLIB' with the commands:

```
>STADLIB=/usr/local/staden/lib
>export STADLIB
```

Note: **pISTil** uses its own **Pregap4** configuration file 'pregap4_pistil.config' provided in the **pISTil** directory. All settings can be changed to specify their own parameters.

9. Phred software - http://www.phrap.org/phredphrapconsed.html

The **Phred** software reads DNA sequencing trace files, calls bases and assigns a quality value to each called base.

pISTil has been tested for the 0.020425.c version of **Phred**.

Install **Phred** as described in the INSTALL file that comes with the **Phred** software. Make sure to set 'PHRED_PARAMETER_FILE' environment variable correctly. It should point to the phredpar.dat **Phred** parameter file that comes with **Phred**.

Since the **Phred** base calling depends on the correct identification of chromatogram 'source', please check if your **Phred** parameter file includes these lines:

```
"DT3730POP7{ET}.mob"      terminator     energy-transfer      ABI_3700
"DT3730POP7{BDv3}.mob"     terminator    big-dye           ABI_3700
```

Get **Phred** from bge@u.washington.edu (Brent Ewing).

10. JDK -- http://www.sun.com

To view trace files on the web, the **pISTil** interface uses **BMC TraceViewer** (available from Baylor College of Medicine: http://www.hgsc.bcm.tmc.edu/downloads/software/trace_viewer/index.html), a Java applet that allows you to see DNA sequencing traces. The **BMC TraceViewer** source files are included in the **pISTil** source code. You just have to check that the **JDK** is installed.

11. csh shell

A shell is a program which provides a user interface. With a shell, users can type in commands and run programs on a Unix system. The **C shell** was written by Bill Joy at the University of California at Berkeley. Check if you have the **C shell** in your Unix system or install it.

III. INSTALLATION AND CONFIGURATION

1. Downloading and unzipping pISTil

The home page of the **pISTil** project is available on the Sourceforge at http://sourceforge.net/projects/pistil.

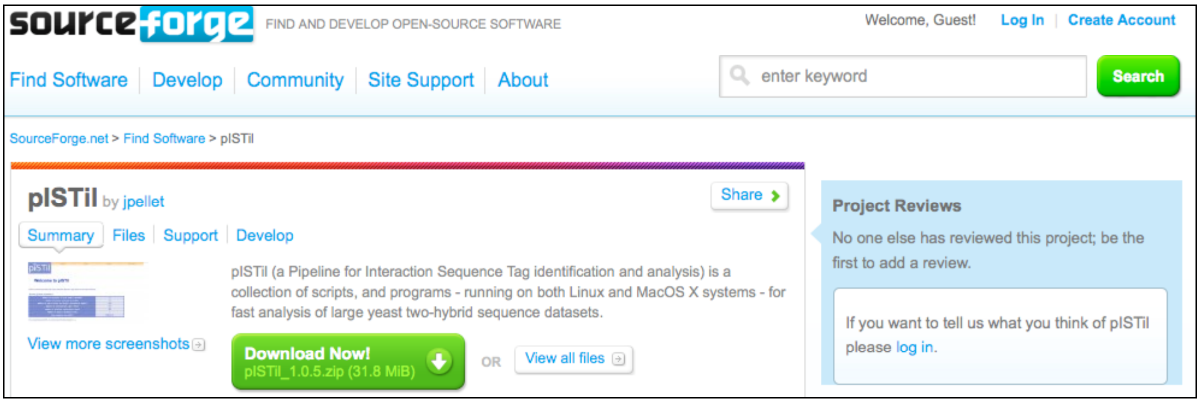

To download the **pISTil** sources, click the Download link.

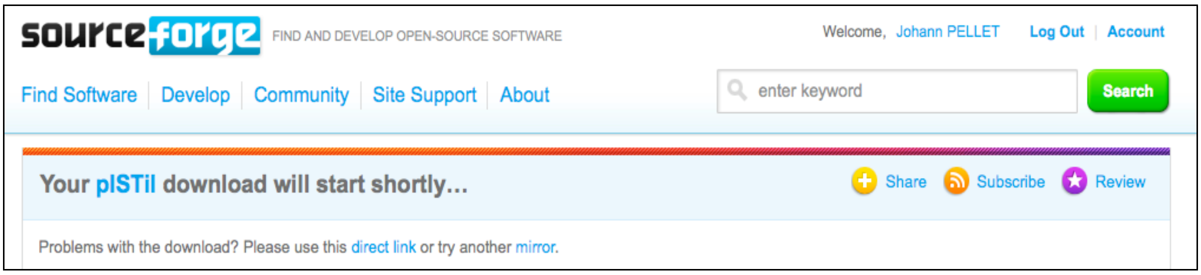

The download of the last release of **pISTil** will start.
You can also browse **pISTil** releases by clicking on the "Files" link:

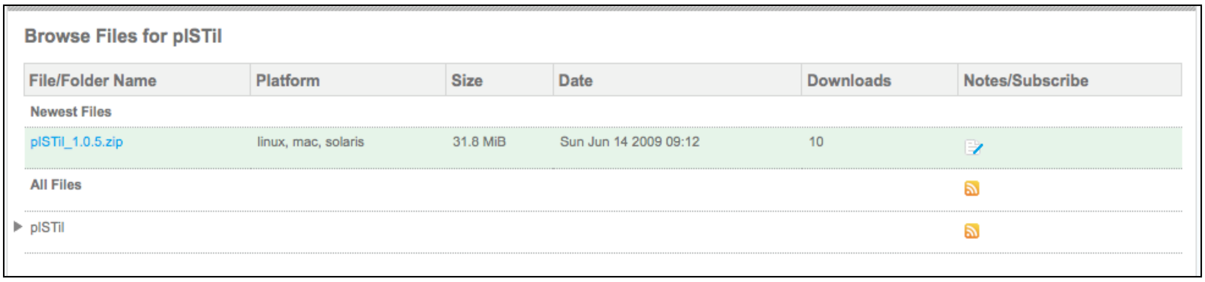

Note: - You don't need to create a Sourceforge account to download **pISTil**.

Unzip and move the **pISTil** directory to a subdirectory in your main web directory:

- For **MAMP** users, the standard web directory is /Applications/MAMP/htdocs.
- For Linux users, the standard web directory varies, but generally takes the form of /var/www/html.

2. Creating the pISTil database:

pISTil uses a single database with 16 tables.
The "create_database.csh" script in the pISTil/db folder creates automatically the database.

You must use a **PostgreSQL** account, which has all privileges. If you don't have it, use the following command in your shell to create the **pISTil** user 'IST_user' with password 'istdb':

>createuser IST_user -d -l -W -P

- At the questions:

>Shall the new role be a superuser? (y/n)

- You can answer no 'n'.

>Shall the new role be allowed to create more new roles? (y/n)

- You can answer no 'n'.

>Password:

- Write the password, for example 'istdb'.

Note: Depending on your work environment, the password can be requested at the beginning.

Now you can launch the **csh** script in the pISTil/db directory to create the **pISTil** database. 'create_database.csh' needs two arguments: the first one is the name of the database (ex: 'pistil'), the second one is the user of the database (ex: 'ist_user'). To execute the **csh** script go in the pISTil/db directory and launch the following command:

>csh create_database.csh pistil ist_user

Note: - In the example below, we use 'pistil' for the name of the database, and 'ist_user' for the user name. However you can use the database and user names you want.
- This script will try to drop the database given in argument before starting to create it.

Now you have the **pISTil** database installed with, by defaults, some data used for the analysis of the HCV dataset in 5 tables (see section IV.3).

Note: - The data for 'method', 'midb' and 'reference' tables come from PSI-MI (http://www.psidev.info/).
- For more information about the **pISTil** tables, please see Annex 2.

3. Setting up the pISTil configuration file

pISTil uses a central configuration file named "config_analyse.pm" that contains variables and settings that can be customized. It is localized in the **pISTil** root directory.

- You must configure each variable before using it:
  - **dbname**: name of the database you created for **pISTil**.
  - **dbhost**: name of the **PostgreSQL** server.
  - **dbuser**: user that has access privileges for the **pISTil** database.
  - **dbpass**: password for that user.

- **path_to_pregap_config**: location of the **pregap** config file used by **pISTil**.
- **temp_dir**: some of the scripts need some scratch space. **pISTil** will create this temporary directory in the **pistil** root directory.
- **path_to_databank_pattern**: central location of the **BLAST** databases used to search and localize pattern before cDNA. See Annex 7 for explanation about this pattern.
- **path_to_databank_blastX**: central location of the **BLAST** databases used to identify ISTs.
- **MI_method**: PSI-MI identifier for interaction detection method.
- **phred_arg**: **Phred** processing options. Value can be 'nocall' if you want to disable **Phred** base calling and to set the current sequence to the ABI base calls that are read from the input files. If you want to set trimming error probability, value can be for example '-trim_cutoff 0.01'. The default value is 0.05. Please read more about trimming in the **Phred** documentation.
- **dataset_dir**: directory where you placed the zip file containing your data to analyse.
- **regex_plate**: regular expression for pulling out the plate name.
- **regex_location**: regular expression for pulling out the well location.
- **save_BLASTN**: yes ('y') or no ('n') for saving or not BLASTN results in a file.
- **save_BLASTX**: yes ('y') or no ('n') for saving or not BLASTX results in a file.
- **log_file**: yes ('y') or no ('n') for keeping or not a log file.

Note: To see how to configure the "config_analyse.pm" file for the HCV datasets analysis, please see Annex 3.

- About regular expression: A regular expression (also "regex") is a string that is used to describe or match a set of strings according to certain syntax rules. You must specify two regular expressions to define the plate name and the well location compared to the name of traces. If you are not familiar with regex rules, you can find a short help in the configuration file.

Example with this trace name: HCV15_1_96 -A01-Y2H_AD-9
If we 'translate' this name in regex form:

Name:	HCV15_1_96	-	A	0	1	-Y2H_AD-9
Regex:	^\w+	\-	\w	\d	\d	.*

We define the plate name like **'HCV15_1_96'**. To match it, we use '()':

Name:	HCV15_1_96	-	A	0	1	-Y2H_AD-9
Regex:	^(\w+)	\-	\w	\d	\d	.*

The well location is **'A01'**:

Name:	HCV15_1_96	-	A	0	1	-Y2H_AD-9
Regex:	^\w+	\-	(\w	\d	\d)	.*

Note: Your trace file names must be similar in one plate to work with one regex. Indeed if you have one chromatogram file like 'HCV15_1_96-A01-Y2H_AD-9' and the second one 'HCV15_1_96_A02-Y2H_AD-9', it will not work with the regex '^(\w+)\-\w\d\d.*'.
So you have two options: change the name of the trace file or find a regex that works with both, like '^(\w+)[\-_]\w\d\d.*'.

- About **Phred** processing options: **Phred** can automatically remove low-quality base calls from the start and the end of DNA sequences, a process called "trimming" or "clipping". When generating trimmed output files, you will loose bases at the start and the end of sequences, so trimming should be used with care. If you plan to generate trimmed sequences, you may first want to experiment different cutoff scores to see which setting works better for you. (See Annex 9).

4. Downloading and creating the BLAST databases:

pISTil relies on protein sequence databases to analyse the screening data. You have to use a sequence database referenced in the PSI-MI 2.5 ontology (see Annex 6). Each database has its repository in the pISTil/localdb directory.

For instance you can download NCBI and ENSEMBL flat files from:

- **NCBI**: ftp://ftp.ncbi.nih.gov/blast/db/FASTA/ for GenBank database.
- **Ensembl**: http://www.ensembl.org/info/data/ftp/index.html for Ensembl database.

Move the downloaded file in the fasta format to pISTil/localdb/ddbj-embl-genbank/ for the GenBank database or to pISTil/localdb/ensembl/ for the Ensembl database.

You must then use this file to construct the index for the **BLAST** database by using the '**formatdb**' program from NCBI. In the following example, **formatdb** is used to construct the **BLAST** database called 'Homo_sapiens.NCBI36.50.pep.all' from the fasta file 'Homo_sapiens.NCBI36.50.pep.all.fa' containing multiple proteic sequences:
In the directory pISTil/localdb/ensembl/ type:

> formatdb -p T -o -i ./Homo_sapiens.NCBI36.50.pep.all.fa

Note: - Download and create the database may take several minutes depending both on your internet connection and your processor speed
- If you want use your own database which is not referenced by PSI-MI (see Annex 6), move your fasta file into pISTil/localdb/other/

5. Creating the pattern BLAST database

pISTil relies on **BLASTN** to accurately locate the beginning of cDNA insert by making use of a database of vector construct sequences (see Annex 11). Thus, according to the cDNA library screened, **pISTil** will align the vector sequence before the cDNA and thus will retained only cDNA sequence for protein assignation. Accurate localization of the vector construct is also crucial to characterise cDNA that were encoded "in-frame" into the two-hybrid system (or other systems, according to the fusion protein).

To insert library and vector data into the database, you have to use the **pISTil** interface (see section III.8).

6. Configuring "the bait parameter file"

The file 'define_bait' is located by default in the **pISTil** root directory. This file is used to identify baits present in each of the 96 wells of a plate.

To configure this file for the **pISTil** software you must give: the first then the last well where one bait is present, the product of this bait and optionally its database accession number and its PSI-MI database identifier. The values are separated by tabulations.

Example:

First well	Last well	Bait product	Bait proteinid	PSIMI database id
A01	A01	NS3	CAB46677	0475
A02	A04	NS4	CAB46677	0475

In this example, in A01, the bait is NS3, and from A02 to A04 the bait is NS4, both from Hepatitis C virus (taxon=11103). The GenBank accession for these both bait products is CAB466677, a polyprotein. The PSIMI database identifier for GenBank is 0475.
'Bait proteinid', 'PSIMI database id' are required if you are going to export protein-protein interaction lists to PSI-MI format. 'Bait proteinid' is the identifier of the bait according to the database described in the following field. 'PSIMI database id' is the PSI-MI identifier for this database (See Annex 6 to choose the right identifier). If you use a personal database to identify your bait, interactions involving this bait won't be exportable in PSI-MI format.

If you have several plates for a single project, you can analyse all traces at once. However you must configure the bait parameter file by specifying the plate name before description of the plate content.

Example:

First well	Last well	Bait product	Bait proteinid	PSIMI database id
--HCV15_1_96				
A01	H12	NS3	CAB46677	0475
--MARIE1				
A01	H12	NS4	CAB46677	0475

In this example, pISTil will analyse two plates, 'HCV15_1_96' with NS3 in all wells, and 'MARIE1' with NS4 in all wells.

Note: - Don't forget to write '--' before the plate name.
- The plate name must be identical to the one extracted from the regex (section III.3)
- Don't change the configuration file format to identify baits.

7. Setting up the pISTil interface

The **pISTil** web interface (ex: http://localhost/pISTil/www) provides a powerful and user-friendly way to query and to navigate throughout the **pISTil** results.

First, you need to fill up a configuration file named 'config_www.inc' in the pISTil/www/inc directory. This file contains many variables and settings that can be customized:

- **$HOST_NAME**: name of the **PostgreSQL** server.
- **$DATABASE_NAME**: name of the database you created for **pISTil**.
- **$DATABASE_USER**: user that has all access privileges for the **pISTil** database.
- **$DATABASE_PASSWORD**: password for that user.
- **$LOCAL_DIR**: location of the **pISTil** directory which contains all the data and the scripts for the interface.
- **$FORMATDB_EXEC**: absolute path to **formatdb** to use when formatting the blast pattern database. Type *which formatdb* in your terminal to know its path.

- **$LOCALDB_PATH**: absolute path for the 'localdb' subdirectories in the **pISTil** directory.

Note: To see how to configure the 'config_www.inc' file for the HCV datasets, please see Annex 5.

8. Edit library and vector data

To insert or remove library or vector data in the **pISTil** database, use the **pISTil** web interface.

- In the **pISTil** home page, select "Library screening" from the "Information" drop-down menu. This page shows you all vectors and libraries already inserted in the database.
- When you want to insert a new library you need to specify a vector. So you must first insert a vector if it's not already in the database.

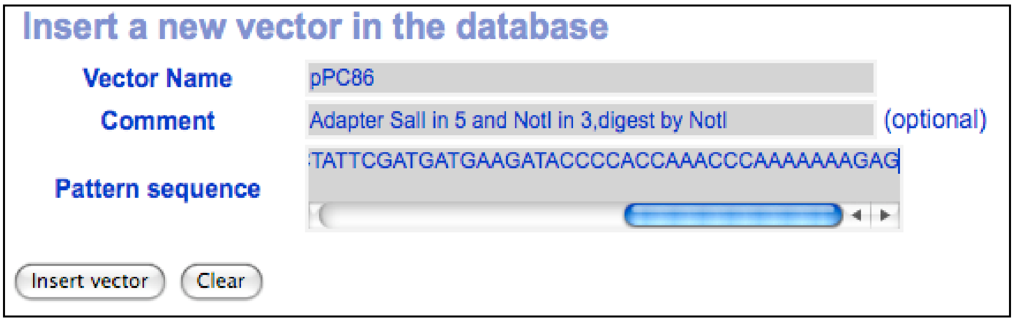

- To insert a vector, fill out the vector form, and click the button insert.

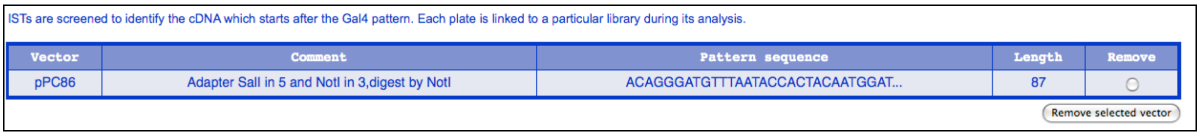

ISTs are screened to identify the cDNA which starts after the Gal4 pattern. Each plate is linked to a particular library during its analysis.

Vector	Comment	Pattern sequence	Length	Remove
pPC86	Adapter SalI in 5 and NotI in 3,digest by NotI	ACAGGGATGTTTAATACCACTACAATGGAT...	87	○

Remove selected vector

Note: When you insert a new vector, the **pISTil** interface will automatically format the pattern database.

After that, the new vector will appear in the vector field of the library form.

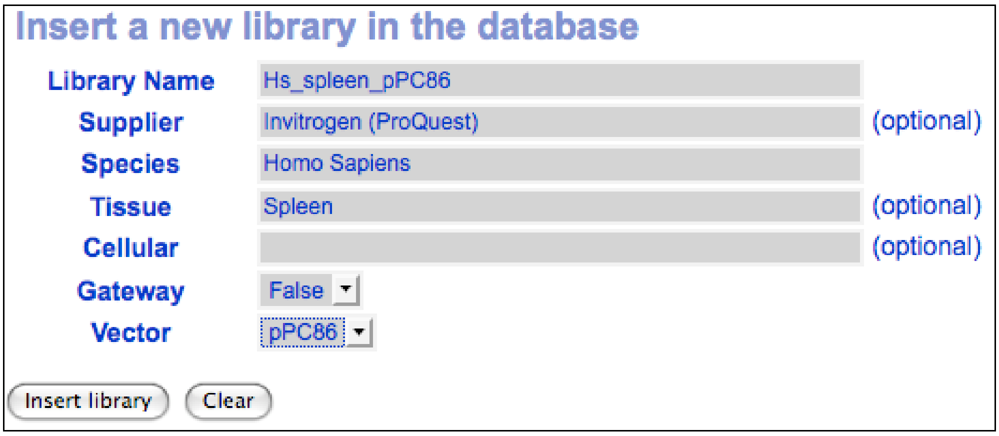

- To insert a library, fill out the library form, and click the button insert.

- When you want to remove a vector or a library, select it and click to the remove selected vector or library button. Note than if you delete a vector, the database server also deletes any libraries associated with that vector.

IV. RUNNING pISTil:

1. Quick start:

Running **pISTil** is very simple once the configuration files have been set on.

The default command in you shell is:

>perl ist_analyse.pl <zip_file>

Input zip file containing all the traces from one or more plates of the same project.

Note: The zip file is one of the archive file in pISTil/dataset directory.

2. Running with your own bait parameter file:

If you have more than one configure file to define the baits or if you change its name 'define_bait', run **pISTil** with a second argument.

>perl ist_analyse.pl <zip_file> <your_config_bait_file>

3. Example with the two HCV datasets:

In this example, we analyse two datasets from I-MAP team experiments (de Chassey B, Navratil V, Tafforeau L *et al.*, Hepatitis C Virus infection protein network. Molecular Systems Biology 4:230, 2008).

These two datasets are distributed with **pISTil** and already in the pISTil/dataset directory. HCV.zip contains 96 trace files from yeast two-hybrid screening against a *Homo sapiens* spleen library. HCV2.zip contains 96 traces from two hybrid screening against a *Homo sapiens* fetal brain library.

We consider that the **pISTil** database has already been created as described in section III.2, using 'pistil' as database name, 'ist_user' as **PostgreSQL** user and 'istdb' as password. Please adapt the corresponding variables in the "config_analyse.pm" and "config_www.inc" files if you have used other parameters.

As a case study, we have analysed all chromatograms to annotate ISTs based on the RefSeq protein database (NCBI Reference Sequence (RefSeq): a curated non-redundant sequence database of genomes, transcripts and proteins. Pruitt KD, Tatusova, T, Maglott DR Nucleic Acids Res 2007 Jan 1;35(Database issue):D61-5).

Download the 'human.protein.faa.gz' file from ftp://ftp.ncbi.nih.gov/refseq/H_sapiens/mRNA_Prot/ into the pISTil/localdb/refseq/ directory.

To unzip the compressed file, execute in your terminal window:
>gunzip human.protein.faa.gz

Now we have to format this file to construct the index for the **BLAST** database by using **formatdb** program from NCBI.

In the directory pISTil/localdb/refseq/ execute this command:
> formatdb -p T -i ./human.protein.faa –o –n refseq_human_prot

Please ensure to correctly:

- configure the config_analyse.pm file (see Annex 3) localized in the pISTil directory
- configure the config_www.inc file (see Annex 5) localized in the pISTil/www/inc directory

For the demo, library and vector data were already integrated into the **pISTil** database, so you don't have to insert them for this example. Hence, in the library and vector page in the web interface, you can see these vector data:

Vector	Comment	Sequence	Length
pPC86	Adapter SalI in 5 and NotI in 3,digest by NotI	ACAGGGATGTTTAATACCACTACAATGGAT...	87

And these library data:

Libary	Supplier	Species	Tissue	Cellular	Gateway	Vector
Hs_fetal_brain_pPC86	Invitrogen (ProQuest)	*Homo Sapiens*	Fetal Brain	-	f	pPC86
Hs_spleen_pPC86	Invitrogen (ProQuest)	*Homo Sapiens*	Spleen	-	f	pPC86

Let's start the first analysis with HCV.zip.

First we check the file 'define_bait' localized in the pISTil directory.
All baits in this plate are the same viral protein, NS3. The protein GenBank accession number is ' CAB46677' and the PSI-MI identifier for GenBank is MI:0475.

First well	Last well	Bait product	Bait proteinid	PSIMI database id
A01	H12	NS3	CAB46677	0475

- Now we can run the pipeline:

>perl ist_analyse.pl HCV.zip

Your prompt shell shows you:

```
##################################################################
Check your pISTil configuration file...... please wait
All parameters in the configuration file seem to be good.
Check your Phred argument.Please wait
Phred will be executed without basecalling!

#####################################################:
Before starting, pISTil needs some information about the project to analyse
List of projects in the pISTil database:
No project in the pISTil database
Choose a project or create a new one (0)
Project identifier=
```

Write '0' to create a new project.

```
Project identifier= 0
You decide to create a new project:
Project name:
```

Choose a project name and a description:

```
Project name: Hepatitis C virus
Project description: Screening from the IMAP team
All the data needed to create this new project is now recorded

------------------------------------------------:
Choose the library used for the screen.
List of libraries in the pISTil database.
1	|	Hs_spleen_pPC86 |	Homo Sapiens	|	Spleen |	|	pPC86
2	|	Hs_fetal_brain_pPC86	|	Homo Sapiens	|	Fetal Brain	|	|	pPC86
Choose a library identifier:
Library identifier:
```

You must select the appropriate library for the analysis. This first dataset comes from a screen against the *Homo sapiens* spleen library, identified by '1'.

```
Library identifier= 1
pISTil will use the library identifier 1
Unzip your zip file and check your raw files
Unzip HCV.zip

*CHECK ALL RAW FILE NAMES BEFORE STARTING THE PIPELINE
Is your regex is correct for HCV15_1_96-A01-Y2H_AD-9?
Plate name=HCV15_1_96, Location=A01.
Choose yes (y) or no (n):
```

pISTil analyses all traces files and tests you regex. If it's correct, write 'y' for yes:

```
Choose yes (y) or no (n):y
pISTil has detected 1 plate(s)
Results: 96 raws checked and validated.
Check plate HCV15_1_96.
It's a new plate for this project.
####################################################:
Now pISTil running the analysis at Sun Jan  4 22:52:8 2009
……
End  pISTil at Sun Jan  4 22:56:49 2009
----------------------------------------------------
Blast statistics:
Number of corrections = 45
Number of BlastX hits=93
Number of BlastX no hits=3
----------------------------------------------------
Do you want insert your data on the pISTil database?
Yes (y) or No (n)
```

At the end of the **pISTil** pipeline, you have the choice to insert automatically all results in the **pISTil** database, or to do it manually using sql files generated during the analysis.

```
Yes (y) or No (n)
y
INSERT 0 1
INSERT 0 1
You have inserted your data. pISTil success.
```

At this step, we have analysed the first dataset. Now we have to change two parameters before starting with the second one, named 'HCV2.zip'. First we must be sure that all regex in the "config_analyse.pm" file are correct according to trace file names. Here, regex are the same than for the first analysis. Secondly, we must change the "define_bait" file and configure it according to the criteria of the second plate.

Here are the lines for the "define_bait" file:

First well	Last well	Bait product	Bait proteinid	PSIMI database id
A01	F12	NS3	CAB46677	0475
G01	H02	NS2	CAB46677	0475
H03	H12	NS3	CAB46677	0475

Now you can launch the **pISTil** pipeline with the second dataset 'HCV2.zip'.

>perl ist_analyse.pl HCV2.zip

```
###################################################################
Check your configuration file...... please wait
All parameters in the configuration file seem to be good.
Check your Phred argument.Please wait
Phred will be executed without basecalling!

######################################################:
Before starting, pISTil needs some information about the project to analyse
List of projects in the pISTil database:
0.....     Create a new one
1    |     Hepatitis C virus    |    Screening from the IMAP team    |    2009-01-04 23:27:13
Choose a project or create a new one (0)
Project identifier=
```

In this tutorial, we analyse this new dataset in the same project than before. So we type '1'.

```
Project identifier= 1
pISTil will use the project identifier 1

-----------------------------------------------:
Choose the library used for the screen.
List of libraries in the pISTil database.
1    |    Hs_spleen_pPC86 |   Homo Sapiens  |   Spleen  |        |   pPC86
2    |    Hs_fetal_brain_pPC86    |  Homo Sapiens  |   Fetal Brain    |        |   pPC86
Choose a library identifier:
Library identifier=
```

We select the appropriate library, identifier '2':

```
Library identifier= 2
pISTil will use the library identifier 2
Unzip your zip file and check your raw files
Unzip HCV2.zip

*CHECK ALL RAW FILE NAMES BEFORE STARTING THE PIPELINE

Is your regex correct for MARIE1-A01-Y2H_AD-96.ab?
Plate name=MARIE1 , Location=A01.
Choose yes (y) or no (n):
```

pISTil asks if your regex is correct:

```
Choose yes (y) or no (n):y
pISTil has detected 1 plate(s)
Results: 96 raws checked and validated
Check plate HCV15_1_96.
It's a new plate for this project.
#######################################################:
Now pISTil running the analysis at Sun Jan  4 23:46:3 2009
```

pISTil analyses your traces and identifies ISTs.

```
End  pISTil at Sun Jan  4 23:53:34 2009
-----------------------------------------------------
Blast statistics:
Number of corrections = 13
Number of BlastX hits=95
Number of BlastX no hits=1
-----------------------------------------------------
Do you want insert your data on the pISTil database?
Yes (y) or No (n)
```

We insert all information in the **pISTil** database.

```
Yes (y) or No (n)
y
You have inserted your data. pISTil success.
```

Note: A summary of the **pISTil** analysis results for the complete HCV dataset is given in Annex 10.

4. Miscellaneous

Running '*perl ist_analyse.pl*' without argument will display **pISTil** error: "Must give a zip file name localized in dataset directory".

Running '*perl ist_analyse.pl --help*' or '*perl ist_analyse.pl -h* ' option will display **pISTil** quick help launch.

Running '*perl ist-analyse.pl --fasta*' or '*perl ist_analyse.pl -f* ' option allows the use of ASCII fasta sequence files instead of chromatogram files. The method of analysis remains the same, without **Phred** extraction and quality analysis.

5. pISTil processing time

Computer configuration:

Machine: MacBook pro 15"
CPU: 2.33 GHz intel Core 2 Duo
Memory: 2Go 667MHz

Dataset	HCV.zip	HCV2.zip
Plate name	HCV15	MARIE1
Number of baits	96	96
Number of ISTs	93	95
CPU time for a plate (sec)	238,77	390,58
CPU time for one IST (median sec)	1,85	3,17

V. pISTil WEB INTERFACE

After pipeline processing of the chromatogram dataset and data insertion into the **pISTil** database, open your web browser and go to the web folder in which **pISTil** is located, for example http://localhost/pISTil/www/.

You should see a welcome page with some global statistics about all analyses run by **pISTil** and a menu to navigate throughout results:

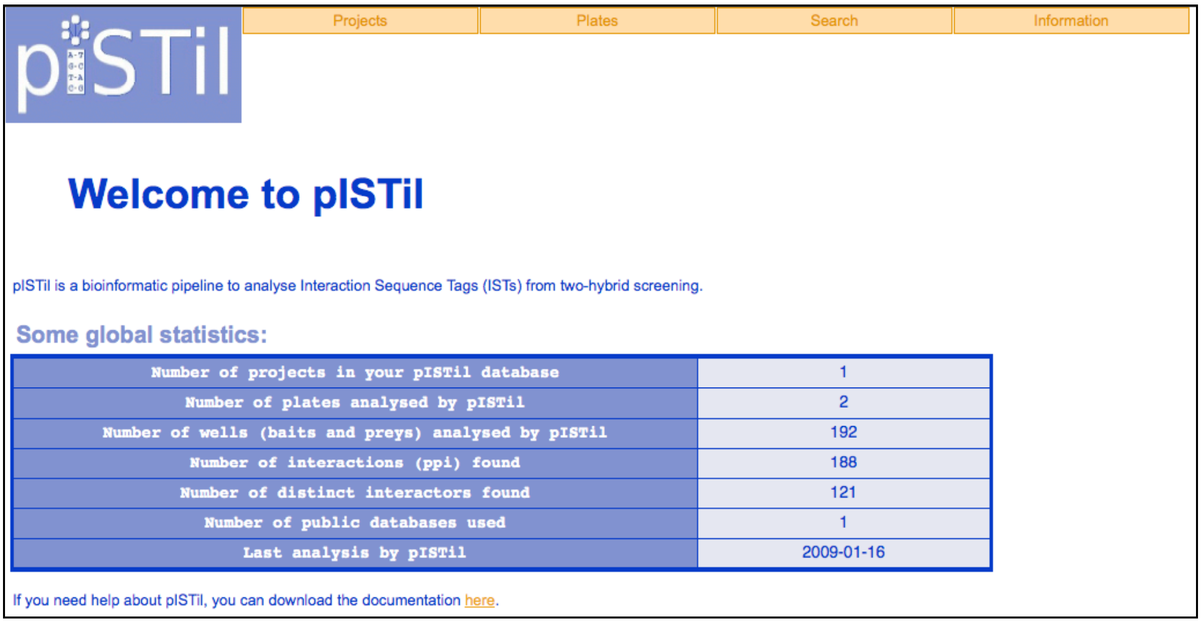

1. Viewing projects

Once projects have been added to the database, they can be browsed using the web menu. A project includes one or more plates of DNA sequences, which have been analysed by **pISTil** software to identify interactors.

To see all projects inserted in the **pISTil** database, use the menu and click on the "Projects" tab.

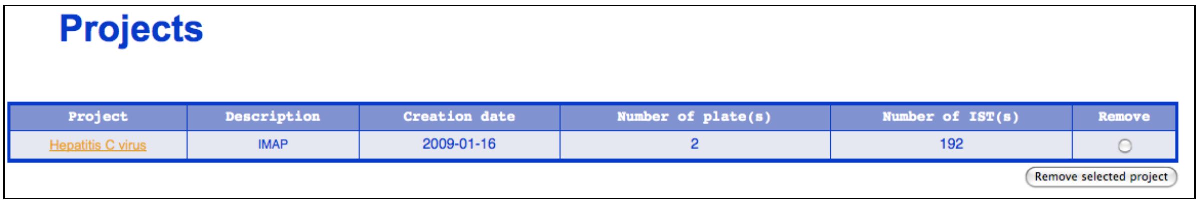

By checking the remove radio button and clicking to the delete button, you can remove a project and all associated information. A confirmation page will appear:

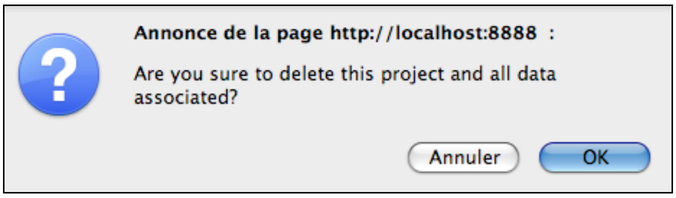

By clicking on a project name, you can access detailed information on the current project including the plates that have been added to this project.

Project information

Project information	
Project	Hepatitis C Virus
Description	IMAP team
Date created	2009-01-16
Plates	2
Baits	2
Wells	192
Interactions	188
Interactors	121
Database	1

Plate	Date created	Preys	Analysis	ISTs
HCV15_1_96	2009-01-16	96	1	93
MARIE1	2009-01-16	96	1	95

By clicking on an analysis link, which corresponds to the number of analysis done for this plate, you can access plate analysis information.

If your plate has been analysed only once:

Plate analysis information

Plate **HCV15_1_96** is analysed **1** times.

Analysis	Phred no base calling	Phred trim	Number of pattern	Number of ISTs	Databank
	True	-	89	93	refseq_human_prot (refseq)

If you have analysed a plate more than one time, here for example the plate "MARIE1" was analysed with two different **BLASTX** databases and different **Phred** parameters:

Plate analysis information

Plate **MARIE1** is analysed **2** times.

Analysis	Phred no base calling	Phred trimming	Number of patterns	Number of ISTs	Databank	Remove
	False	0.01	70	82	Homo_sapiens.NCBI36.50.pep.all.fa (ensembl)	
	True	-	94	95	refseq_human_prot (refseq)	

Remove selected analysis

By clicking on the green arrow, you can access plate information.

2. Viewing plates

You can access plate information using the "Plates" tab from the menu or by clicking on a plate name from a project information page, described below.

Plates

Plate	From project	Last analysis date	Number of analysis	Number of bait(s)	Remove
MARIE1	Hepatitis C virus	2009-01-16	2	96	
HCV15_1_96	Hepatitis C virus	2009-01-16	3	96	

Remove selected plate

If you click on the name of the project, you will be brought to the project information page.

If you click on the name of the plate, you will be brought to the plate information page, which shows you more detailed information about each well on the plate.

If you have analysed a plate more than one time, for example with another **BLASTX** database, you must choose one analysis before seeing all plate information:

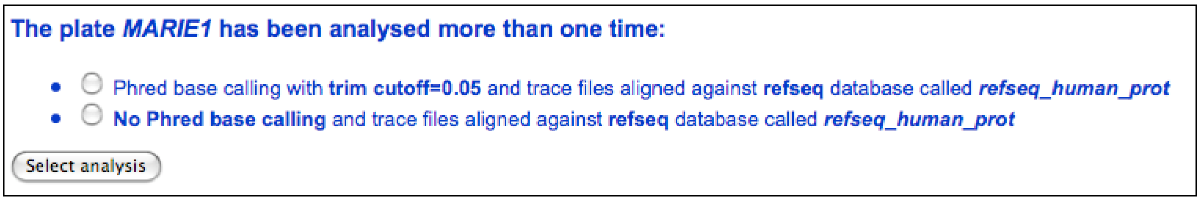

Check one of the analysis and click to the "Select analysis" button.

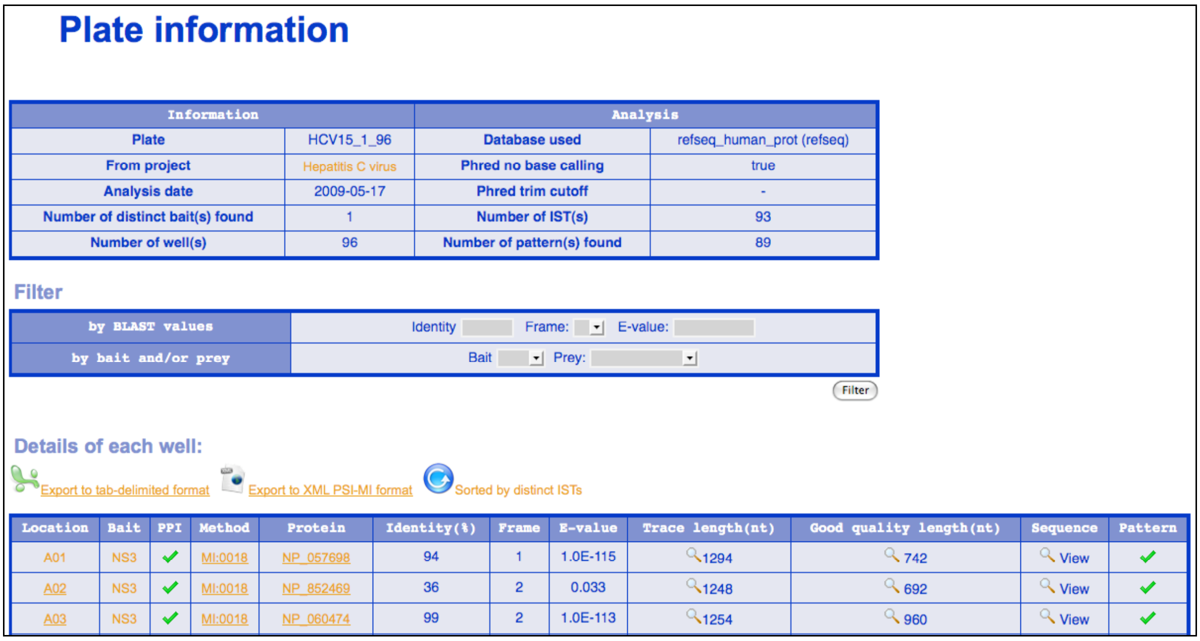

Plate information

Information		Analysis	
Plate	HCV15_1_96	Database used	refseq_human_prot (refseq)
From project	Hepatitis C virus	Phred no base calling	true
Analysis date	2009-05-17	Phred trim cutoff	-
Number of distinct bait(s) found	1	Number of IST(s)	93
Number of well(s)	96	Number of pattern(s) found	89

Filter

by BLAST values	Identity Frame: E-value:
by bait and/or prey	Bait Prey:

Filter

Details of each well:

Export to tab-delimited format Export to XML PSI-MI format Sorted by distinct ISTs

Location	Bait	PPI	Method	Protein	Identity(%)	Frame	E-value	Trace length(nt)	Good quality length(nt)	Sequence	Pattern
A01	NS3	✓	MI:0018	NP_057698	94	1	1.0E-115	1294	742	View	✓
A02	NS3	✓	MI:0018	NP_852469	36	2	0.033	1248	692	View	✓
A03	NS3	✓	MI:0018	NP_060474	99	2	1.0E-113	1254	960	View	✓

The top table lists general information about the plate and the analysis done by the **pISTil** software.
By using the filter table, users can choose a combination of filters to generate different lists. After searching and eventually filtering interactions, you can export the resulting table to tab-delimited format for Excel (or a text editor) by clicking on the "export to tab-delimited format" link (please save the file first, before opening). You can also export the list of chosen interactions to MIMIx PSI-MI format (see section V.5).

The second table lists all of the wells along with their analysis results. Bait and Protein columns include direct links towards public databases according to the 'define_bait' configuration file for baits and the Blast databank used for the IST identification for preys.

You can see the IST sequence corresponding to a well by clicking on the corresponding "View" link.

PISTIL fasta IST sequence:

```
>HCV15_1_96-A01-Y2H_AD-9;Phred base calling with trim cutoff=0.05;662 bp
GGTGGGTCGACCCACGCGTCCGCCCACGCGTCCGGTTGACCTAACCAATGAAGAAACAAC
TGATTCCACCACTTCTAAAATCAGCCCATCTGAAGATACTCAGCAAGAAAATGGCAGCAT
GTTCTCTCTCATTACCTGGAATATTGATGGATTAGATCTAAACAATCTGTCAGAGAGGGC
TCGAGGGGTGTGTTCCTACTTAGCTTTGTACAGCCCAGATGTGATATTTCTACAGGAAGT
TATTCCCCCATATTATAGCTACCTAAAGAAGAGATCAAGTAATTATGAGATTATTACAGG
TCATGAAGAAGGATATTTCACAGCTATAATGTTGAAGAAATCAAGAGTGAAATTAAAAAG
CCAAGAGATTATTCCTTTTCCAAGTACCAAAATGATGAGAAACCTTTTATGTGTGCATGT
GAATGTGTCAGGAAATGAGCTTTGCCTTATGACATCCCATTTGGAGAGCACCAGAGGGCA
TGCTGCGGAACGAATGAATCAGTTAAAAATGGTTTTAAAGAAAATGCAAGAGGCTCCAGA
GTCAGCTACAGTTATATTTGCAGGAGATACAAATCTAAGGATCGAGAGGTTACCAGATG
TGGTGGTTTACCCAACAACATTGTGGATGTCTGGGAGTTTTTTGGGCAAACCTAAACATT
GC
```

General format for the FASTA sequence header:

>	HCV15_1_96-A01-Y2H_AD-9;	Phred base calling with trim cutoff=0.05;	662 bp
	Trace file name	Phred analysis	Length

- If you click on one of the "good quality length" link, you will see the corresponding quality page:

Color codes:

Good quality sequence:	ACGTAC
Low quality sequence:	ACGTAC

>HCV15_1_96-A01-Y2H_AD-9 usable length = 1294 good length = 742

GGGGTAAACC	CCACCATTGT	GATGATGTAT	ATAACTATCT	ATCTCGATGA
08 08 08 08 08 08 08 12 12 12	10 09 10 08 08 10 10 11 10 07	07 08 08 13 09 11 15 16 28 59	39 40 40 48 40 37 37 37 37 18	17 09 08 08 12 13 37 28 24 21
TGAAGATACC	CCACCAAACC	CAAAAAAAGA	GGGTGGGTCG	ACCCACGCGT
24 21 24 20 35 35 39 39 42 57	57 48 48 43 43 43 43 43 43 43	43 43 43 43 42 33 32 32 30 30	31 33 39 42 47 47 48 48 48 54	57 57 57 57 57 57 57 59 59 54
CCGCCCACGC	GTCCGGTTGA	CCTAACCAAT	GAAGAAACAA	CTGATTCCAC
54 57 59 57 57 57 57 54 54 54	54 47 47 43 43 39 43 39 57 46	54 46 46 44 46 43 43 43 35 35	32 34 38 43 43 44 43 48 48 52	57 52 57 57 57 57 57 57 57 57
CACTTCTAAA	ATCAGCCCAT	CTGAAGATAC	TCAGCAAGAA	AATGGCAGCA
57 57 57 57 57 57 57 57 54 54	54 59 57 52 43 43 43 57 57 57	52 52 43 44 43 57 57 57 57 57	59 44 43 33 33 43 44 59 59 57	57 57 57 57 52 57 52 52 52 59
TGTTCTCTCT	CATTACCTGG	AATATTGATG	GATTAGATCT	AAACAATCTG
59 59 59 57 59 59 59 59 52 52	52 57 57 57 57 52 52 52 52 52	62 62 62 62 62 62 62 62 62 62	62 62 62 62 43 43 62 62 62 62	62 62 62 62 62 62 62 44 44 35
TCAGAGAGGG	CTCGAGGGGT	GTGTTCCTAC	TTAGCTTTGT	ACAGCCCAGA
28 28 32 32 52 62 62 62 62 52	52 52 52 62 62 62 62 62 62 62	62 62 62 52 52 62 62 62 62 62	44 62 43 43 39 43 44 62 52 52	52 62 62 62 43 43 43 39 43 39

This HTML page shows Fasta and colour-coded sequence with quality values assigned by **Phred**. During quality analysis, **Pregap4** calculates the average confidence level for a sliding window. The low quality regions (at the start and end of the sequence) are in red.

Note: to compare **Phred** fasta extraction with or without base calling, see Annex 9.

If you click on one of the PSI-MI interaction detection method, you will see the corresponding method page description:

PSI-MI interaction detection method

MI	Name	Description	PMID
MI:0018	two hybrid	The classical two-hybrid system is a method that uses transcriptional activity as a measure of protein-protein interaction. It relies on the modular nature of many site-specific transcriptional activators (GAL 4) , which consist of a DNA-binding domain and a transcriptional activation domain. The DNA-binding domain serves to target the activator to the specific genes that will be expressed, and the activation domain contacts other proteins of the transcriptional machinery to enable transcription to occur. The two-hybrid system is based on the observation that the two domains of the activator need to be non-covalently brought together by the interaction of any two proteins. The application of this system requires the expression of two hybrid. Generally this assay is performed in yeast cell, but it can also be carried out in other organism.	10967325

If you click on one of the location link, you will see the corresponding protein-protein interaction (ppi) page (see section III.3).

If you click on "Sorted by distinct ISTs" link, you can sort interactions by the frequency of observation and as before you can apply multiple filters.

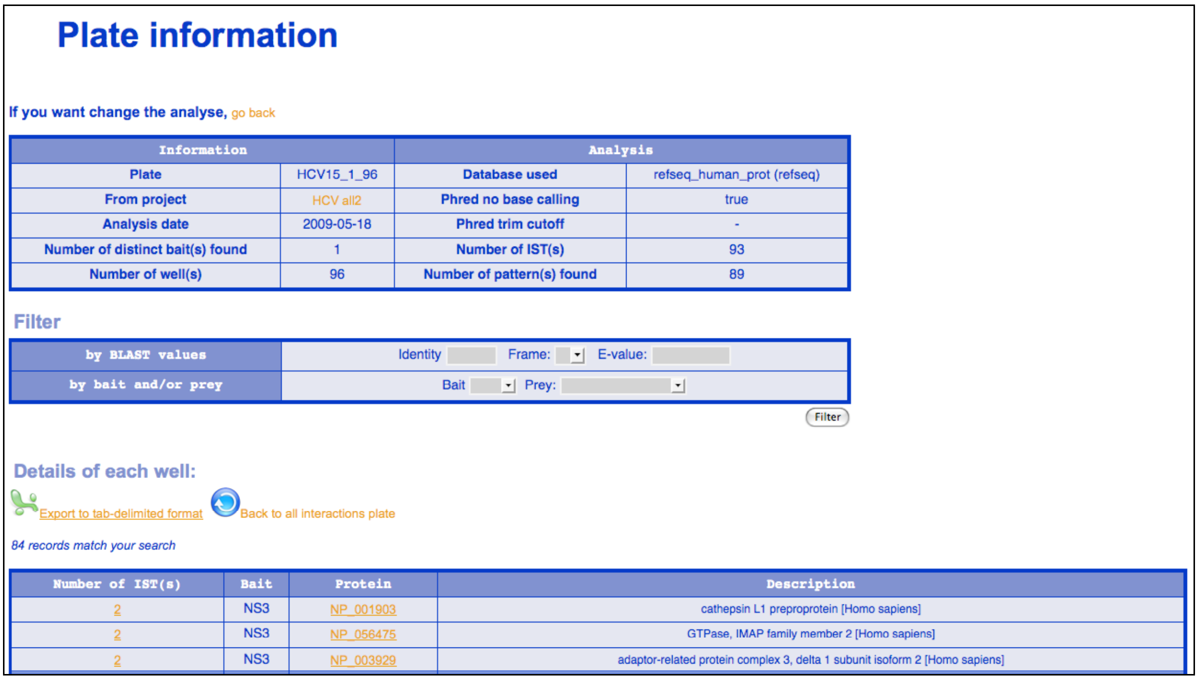

Plate information

If you want change the analyse, go back

Information		Analysis	
Plate	HCV15_1_96	Database used	refseq_human_prot (refseq)
From project	HCV all2	Phred no base calling	true
Analysis date	2009-05-18	Phred trim cutoff	-
Number of distinct bait(s) found	1	Number of IST(s)	93
Number of well(s)	96	Number of pattern(s) found	89

Filter

by BLAST values	Identity Frame: E-value:
by bait and/or prey	Bait Prey:

Filter

Details of each well:

Export to tab-delimited format Back to all interactions plate

84 records match your search

Number of IST(s)	Bait	Protein	Description
2	NS3	NP_001903	cathepsin L1 preproprotein [Homo sapiens]
2	NS3	NP_056475	GTPase, IMAP family member 2 [Homo sapiens]
2	NS3	NP_003929	adaptor-related protein complex 3, delta 1 subunit isoform 2 [Homo sapiens]

3. Viewing protein-protein interaction (ppi)

The ppi page lists all information concerning a specific well:

- Project and plate information:

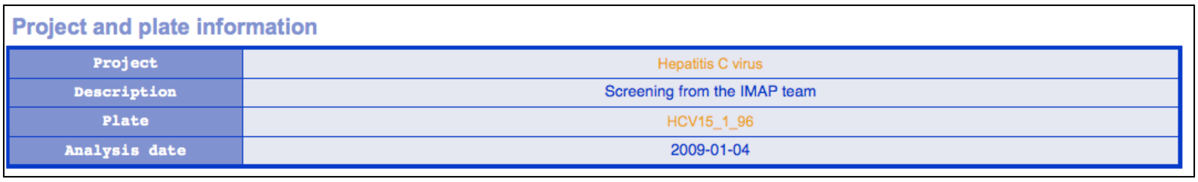

Project and plate information

Project	Hepatitis C virus
Description	Screening from the IMAP team
Plate	HCV15_1_96
Analysis date	2009-01-04

This part shows you the project name, the project description, the plate name for the interaction, and the analysis date.

- Bait information:

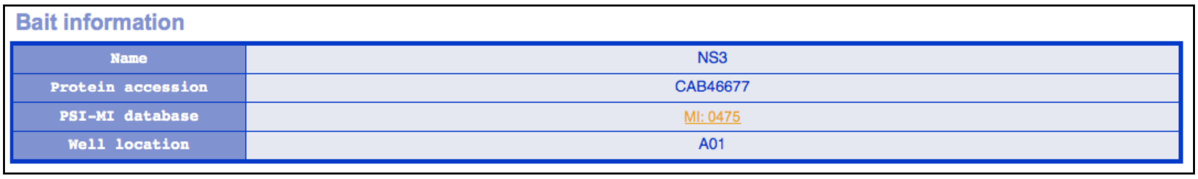

Bait information

Name	NS3
Protein accession	CAB46677
PSI-MI database	MI: 0475
Well location	A01

Here you find the bait name, well location in the plate and occasionally its protein accession number and PSI-MI database identifier. If you click on the PSI-MI link you will be redirected to the PSI-MI databases page (see section V.6).

- Trace information:

Trace information

Raw	HCV15_1_96-A01-Y2H_AD-9
Raw file	Visualize Download
Nucleotid sequence	GGGGTAAACCCCACCATTGTGATGATGTATATAACTATCTATCTCGATGATGAAGATACCCCAC
Quality (bp)	Usable length: 1294; Start good quality: 27; End good quality: 769; Length: 742
Quality analysis	View quality file

The nucleic sequence is the trace sequence extracted by **Pregap4**, which has calculated the start and the end location for the good quality sequence.

If you click on one of the "View quality file" link, you will see the corresponding quality page.

If you click on the 'Visualize' link, you see the chromatogram using the **Trace Viewer** applet:

data/1/phred_scf_dir/HCV15_1_96-A01-Y2H_AD-9..scf

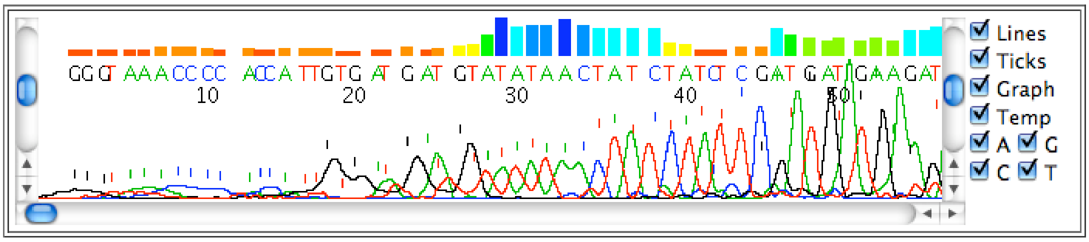

(Visualization performed by BMC Trace Viewer)

If you click on the 'Download' link, you download the trace (in SCF format) on your computer.

- Phred analysis:

Phred analysis

Base calling	No base calling
Phred sequence	GGTGGGTCGACCCACGCGTCCGCCCACGCGTCCGGTTGACCTAACCAATGAAGAAACAACT
Length	1213 bp

The **Phred** sequence is the nucleic sequence used by **BLASTX** to identify IST. This sequence depends on the **Phred** parameter. So if you analyse this trace with two different **Phred** parameters, you can obtained two different IST sequences.

- Pattern information:

Pattern information

<table>
<tr><td>Library</td><td><table><tr><th>Name</th><th>Vector</th><th>Gateway</th><th>Species</th><th>Tissue</th><th>Cellular</th></tr><tr><td>Hs_spleen_pPC86</td><td>pPC86</td><td>False</td><td>Homo Sapiens</td><td>Spleen</td><td>-</td></tr></table></td></tr>
<tr><td>Length pattern</td><td>87 bp</td></tr>
<tr><td>Query</td><td>21 gatgatgtatataactatctatctcgatgatgaagataccccaccaaacccaaaaaaagag 81</td></tr>
<tr><td>Homology (bp)</td><td>|||||||||||||||||||||| ||||||||||||||||||||||||||||||||||||||||</td></tr>
<tr><td>Hit (bp)</td><td>28 gatgatgtatataactatctat-tcgatgatgaagataccccaccaaacccaaaaaaagag 87</td></tr>
<tr><td>Correction</td><td>False</td></tr>
</table>

This table contains all information about the pattern search, according to the vector used in the library. To have an explanation about the "correction" term, please see Annex 11.

- Blast information:

Blast information

Database	Refseq (refseq_human_prot) created: 2009-01-16
Program	BlastX: Compare a nucleotide sequence against a protein database
Alignment	23 QUERY (nucleotid) 682<br>81 HIT (proteic) 296
Hit name	**Protein**: NP_057698
Identity	94 %
E-value	1.0E-115
Frame	1
Hit length	362 aa (1086 bp)
Score	411

This last part of the page gives all **BLASTX** result information. The minimum information about the IST is the protein hit accession number, corresponding to the database used during the analysis. In this case, 94% of query sequence aligned was found identical to the protein NP_057698. This hit is not in frame with the GAL4-AD pattern (Frame=1).

Note: The frame() method of **pISTil** returns 0, 1, or 2 instead of the expected +1, +2, or +3 in BLAST.

4. Search page

Once you have analysed a number of ISTs, it can become difficult to find individual interactor, bait or a special interaction. The **pISTil** web interface proposes thus a search page which is accessible via the "Search" tab in the menu.

Search

by bait	all
and/or by prey	database protein identifier<br>**OR** short protein description
and/or by project	all

Search

You can query interactions found by **pISTil** according to:

- a specific bait: select one bait under the bait drop-down menu.

- a specific prey: specify a protein accession number.
- a short description of a prey.

Alternatively you can filter the result if you select a project using the project drop-down field.

- Example 1: here we search for all interactions of the bait NS2 in the HCV project:

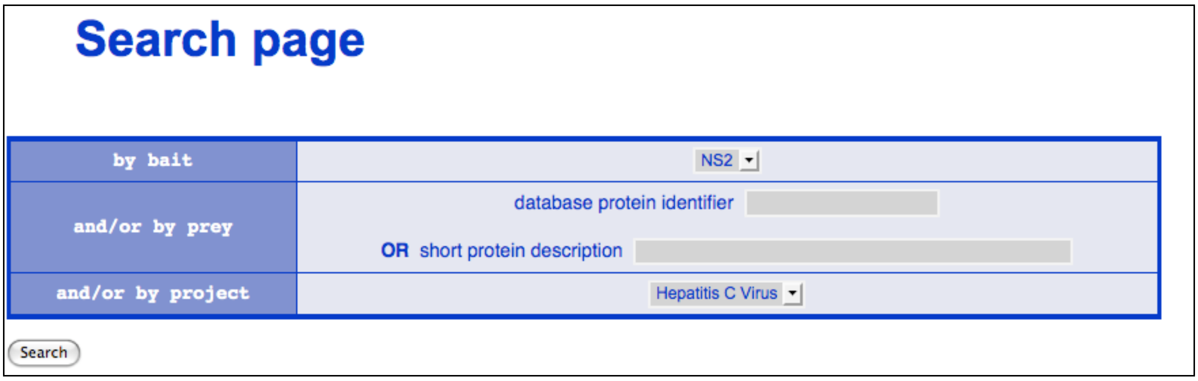

After selecting the correct bait and the HCV project, click on the 'Search' button to see the results:

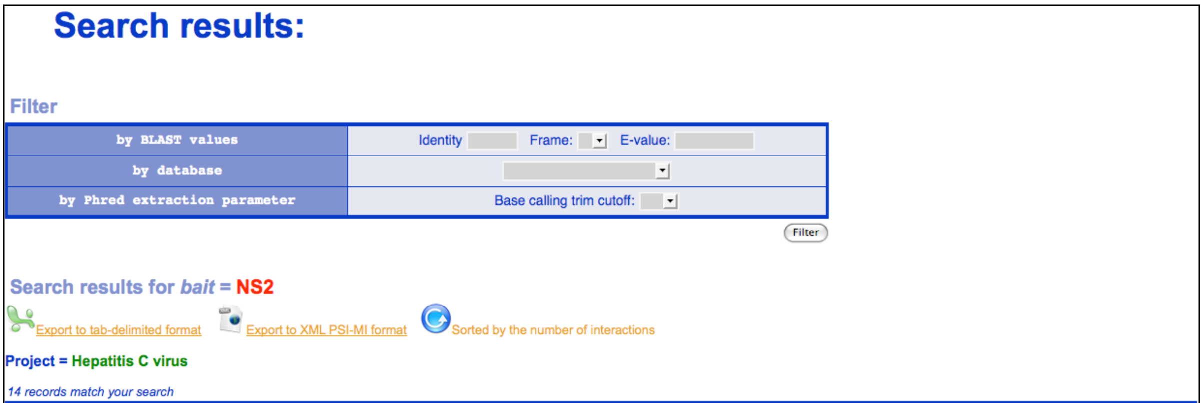

ppi	Bait	Method	Protein	Identity (%)	Frame	E-value	Phred base calling	Library	Description
	NS2	MI:0018	NP_001026884	79	0	1.0E-155	No	Homo Sapiens, Fetal Brain	inverted formin 2 isoform 2 [Homo sapiens]
	NS2	MI:0018	NP_073615	86	0	1.0E-166	No	Homo Sapiens, Fetal Brain	coiled-coil domain containing 21 [Homo sapiens]
	NS2	MI:0018	NP_001026884	89	0	1.0E-170	No	Homo Sapiens, Fetal Brain	inverted formin 2 isoform 2 [Homo sapiens]
	NS2	MI:0018	NP_001026884	80	0	1.0E-162	No	Homo Sapiens, Fetal Brain	inverted formin 2 isoform 2 [Homo sapiens]
	NS2	MI:0018	NP_001026884	84	2	1.0E-121	No	Homo Sapiens, Fetal Brain	inverted formin 2 isoform 2 [Homo sapiens]

We find 14 records. We can filter the results using the filter table by:

- BLAST values: identity and/or frame and/or e-value
- BLAST database
- **Phred** base calling

- Example 2: we want all interactions in frame with the GAL4-AD pattern and with at least 80% identity and an e-value inferior or equal to 1.E-40. So we used the filter table and we click on the filter button after completing the fields as search criteria.

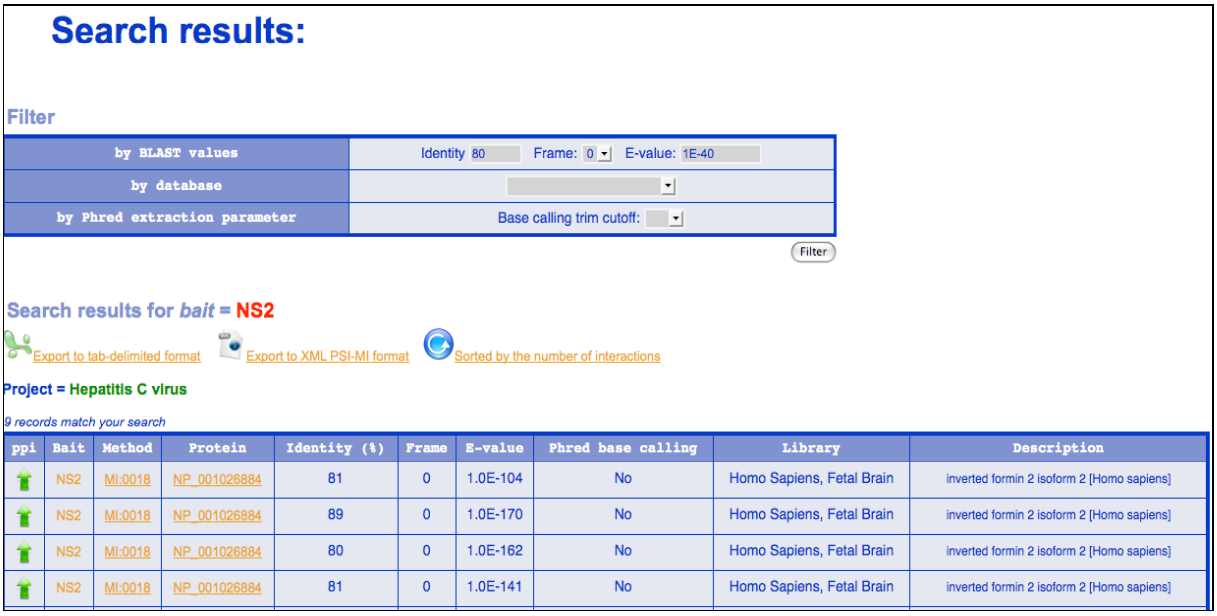

After searching and eventually filtering interactions, you can export the resulting table to tab-delimited format for Excel (or a text editor) by clicking on the "export to tab-delimited format" link (please save the file first, before opening):

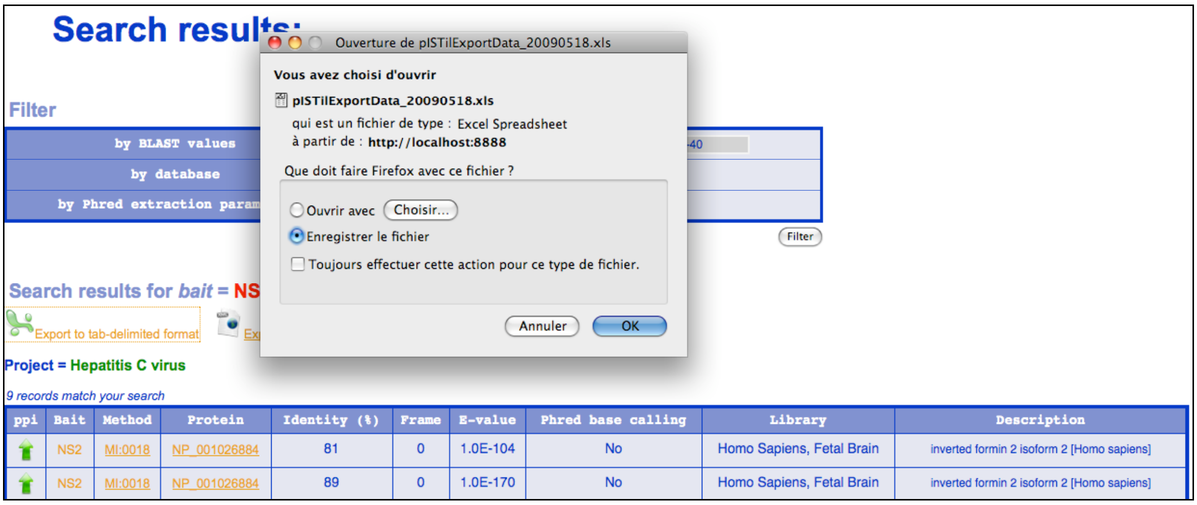

You can also export the list of chosen interactions to MIMIx PSI-MI format (see section V.5).

At last, you can sort the interactions by the number of time they were found (click on the "Sorted by number of interactions" link) as presented in this screenshot:

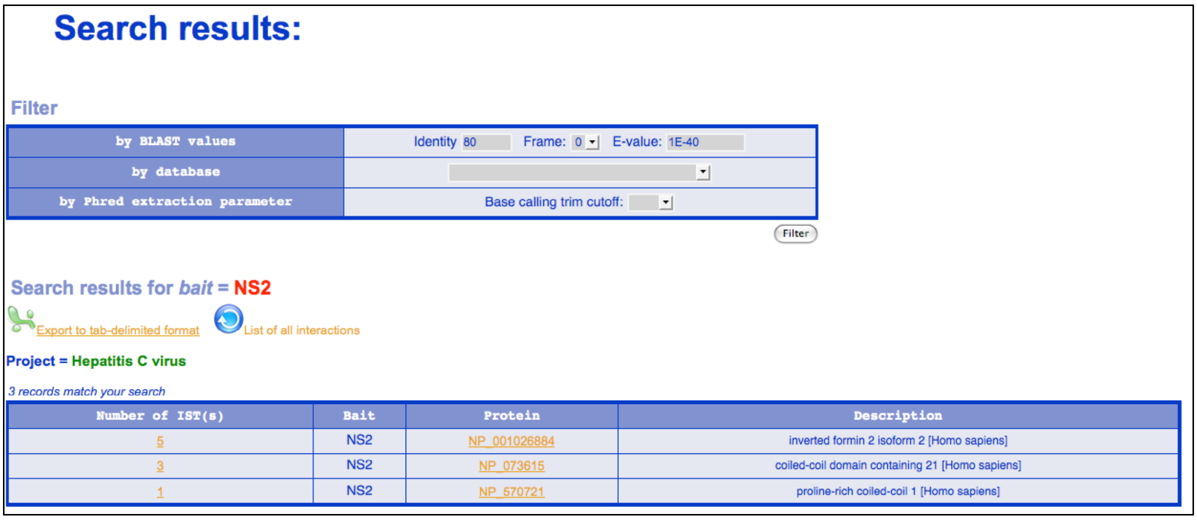

Search results:

Filter

by BLAST values	Identity 80 Frame: 0 E-value: 1E-40
by database	
by Phred extraction parameter	Base calling trim cutoff:

Filter

Search results for *bait* = NS2

Export to tab-delimited format List of all interactions

Project = Hepatitis C virus

3 records match your search

Number of IST(s)	Bait	Protein	Description
5	NS2	NP_001026884	inverted formin 2 isoform 2 [Homo sapiens]
3	NS2	NP_073615	coiled-coil domain containing 21 [Homo sapiens]
1	NS2	NP_570721	proline-rich coiled-coil 1 [Homo sapiens]

Like the precedent search, you can eventually filter interactions and export the displayed table.

The column 'Number of IST(s) ' represents the number of IST found for a given protein-protein interaction, *i.e.* for a given bait and prey protein. If you click on it, you will see the interaction domain:

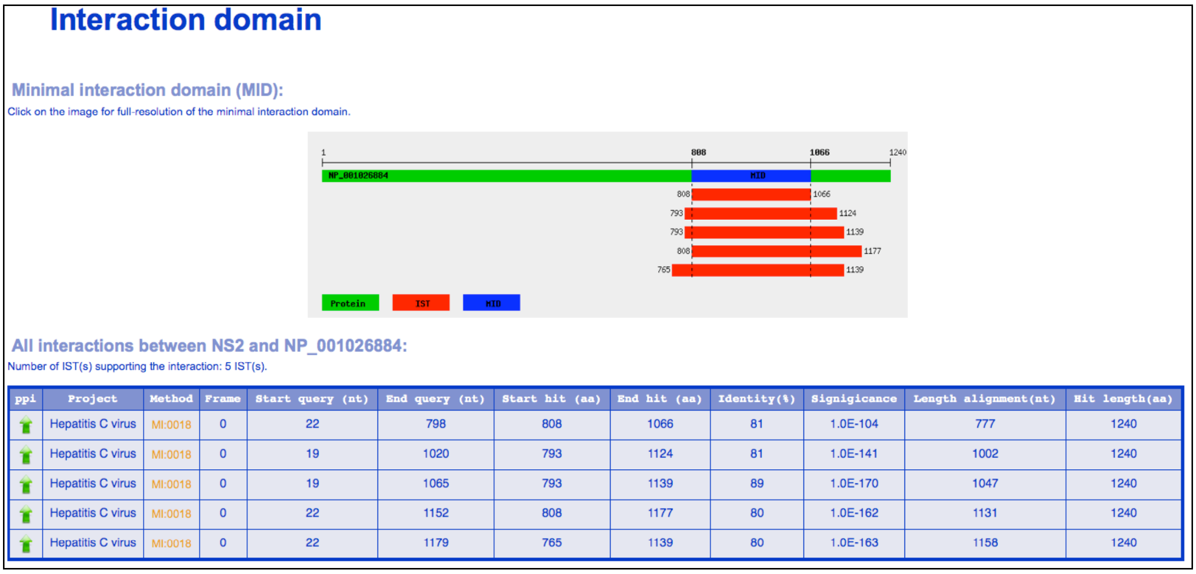

Interaction domain

Minimal interaction domain (MID):

Click on the image for full-resolution of the minimal interaction domain.

All interactions between NS2 and NP_001026884:

Number of IST(s) supporting the interaction: 5 IST(s).

ppi	Project	Method	Frame	Start query (nt)	End query (nt)	Start hit (aa)	End hit (aa)	Identity(%)	Signigicance	Length alignment(nt)	Hit length(aa)
	Hepatitis C virus	MI:0018	0	22	798	808	1066	81	1.0E-104	777	1240
	Hepatitis C virus	MI:0018	0	19	1020	793	1124	81	1.0E-141	1002	1240
	Hepatitis C virus	MI:0018	0	19	1065	793	1139	89	1.0E-170	1047	1240
	Hepatitis C virus	MI:0018	0	22	1152	808	1177	80	1.0E-162	1131	1240
	Hepatitis C virus	MI:0018	0	22	1179	765	1139	80	1.0E-163	1158	1240

The first part of this page is a graphic representation of all ISTs supporting the interaction. We represent in blue the minimal interaction domain (MID), in green the protein and in red ISTs.
The second part is a table with all information about IST alignments.

5. PSI-MI export

MIMIx is the minimum of information required for reporting a molecular interaction experiment, building on the PSI-MI XML v2.5 interchange standard format. You could then thus describe your experimental protein interaction data in a journal article, display it on a website or drop it directly into a public database. The link "export to PSI-MI MIMIx format" leads you to a form, where you have to enter some administrative and experimental informations. The validity of the created file depends on the way you fill in the form.

Please note moreover that :

- Only distinct interactions are considered.
- Only "valid" interactions are integrated in the file, *i.e.* interactions involving proteins referenced in a database listed in PSI-MI 2.5.

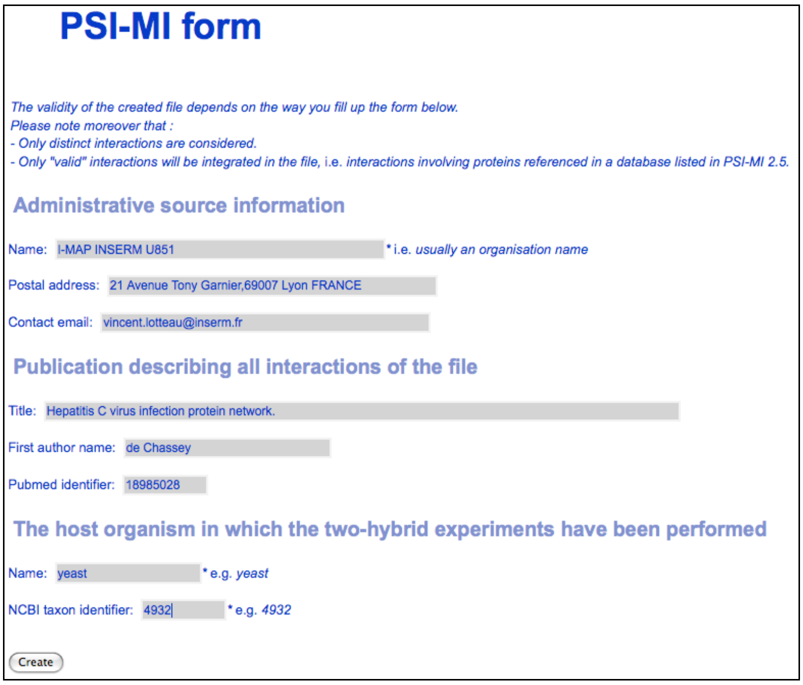

For more informations about PSI-MI and MIMIx, see http://www.psidev.info/index.php?q=node/277 and the reference paper (The minimum information required for reporting a molecular interaction experiment (MIMIx). Orchard et al. Nat Biotechnol. 2007 Aug;25(8):894-8).

When the HTML form is entirely filled, click on the "Create" button. A XML file, placed in the pISTil/www/data directory and named "export_psimi_[date]_[time].xml, is created and filled in according to MIMIx standard with the valid and distinct interactions you have chosen. A new page allows you to see it in your browser or to download it. You can then go back to your search.

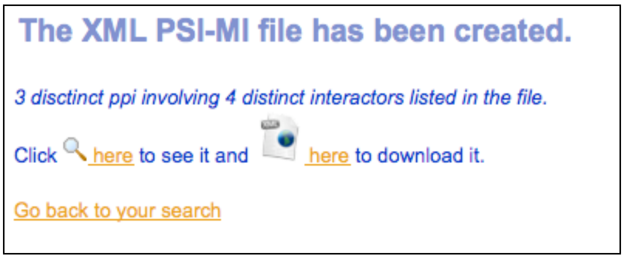

By clicking on the 'see" link you can visualize the XML file in your browser.

```
- <entrySet level="2" version="5" minorVersion="3" xsi:schemaLocation="net:sf:psidev:mi http://psidev.sourceforge.net/mi/rel25/src/MIF25.xsd">
  - <entry>
    - <source releaseDate="2009-01-16">
      - <names>
          <shortLabel>I-MAP INSERM U851</shortLabel>
        </names>
      - <xref>
          <primaryRef db="pubmed" dbAc="MI:0446" id="18985028"/>
        </xref>
      - <attributeList>
          <attribute name="postalAddress">21 Avenue Tony Garnier,69007 Lyon FRANCE</attribute>
          <attribute name="contactEmail">vincent.lotteau@inserm.fr</attribute>
          <attribute name="createdBy">pISTil application, 2009 I-MAP INSERM</attribute>
        </attributeList>
      </source>
    - <interactionList>
      - <interaction id="1">
        - <experimentList>
          - <experimentDescription id="1">
            - <names>
                <shortLabel>de Chassey</shortLabel>
                <fullName>Hepatitis C virus infection protein network.</fullName>
              </names>
            - <bibref>
              - <xref>
                  <primaryRef db="pubmed" dbAc="MI:0446" id="18985028"/>
                </xref>
              </bibref>
            - <hostOrganismList>
              - <hostOrganism ncbiTaxId="4932">
                - <names>
                    <shortLabel>yeast</shortLabel>
                  </names>
                </hostOrganism>
              </hostOrganismList>
            - <interactionDetectionMethod>
              - <names>
                  <shortLabel>two hybrid</shortLabel>
                </names>
              - <xref>
                  <primaryRef db="psi-mi" dbAc="MI:0488" id="MI:0018"/>
                </xref>
              </interactionDetectionMethod>
            </experimentDescription>
          </experimentList>
        - <participantList>
          - <participant id="1">
            - <interactor id="1">
              - <names>
                  <shortLabel>inverted formin 2 isoform 2 [Homo sapiens]</shortLabel>
                </names>
              - <xref>
                  <primaryRef db="refseq" dbAc="MI:0481" id="NP_001026884"/>
                </xref>
              - <interactorType>
                - <names>
                    <shortLabel>protein</shortLabel>
                    <fullName>protein</fullName>
```


Note: you can validate your PSI-MI XML file, exported by **pISTil**, using the **PSI Validator** here: http://www.ebi.ac.uk/intact/validator/psiValidation.jsf

6. Information

To see current **BLAST** databases or to add vectors or libraries in the database, use the "Information" tab in the menu.

If you have already analysed sequences, click on the "Databases" drop down menu, you will see which **BLAST** databases are used:

Databases

	Category	Name	PSIMI	Species	Assembly	Release	Created	Hits
	refseq	refseq_human_prot	MI: 0481	-	-	-	2009-05-17	376
	Ensembl	Homo_sapiens.NCBI36.50.pep.all.fa	MI: 0476	Homo_sapiens	NCBI36	50	2009-05-19	153

Before launching an analysis you must insert in the **pISTil** database vector and library information. Click on the "Library and vector" tab from the "Information" drop down menu (See section III.8 to learn how to insert vector and library data).

If you want to know all information about the PSI-MI databases, click on the "PSI-MI databases" tab from the "Information" drop down menu.

PSIMI database identifier

MI	Name	Description	PMID
0924	camjedb	Camjedb is a comprehensive database for information on the genome of Campylobacter jejuni. http://www.sanger.ac.uk/Projects/C_jejuni/	106882042
0464	cygd	The MIPS Comprehensive Yeast Genome Database (CYGD) aims to present information on the molecular structure and functional network of the entirely sequenced, well-studied model eukaryote, the budding yeast Saccharomyces cerevisiae. In addition the data of various projects on related yeasts are used for comparative analysis. http://mips.gsf.de/proj/yeast/CYGD. http://mips.gsf.de/genre/proj/mpact	14755292
0475	ddbj/embl /genbank	DDBJ EMBL GenBank Nucleotide Sequence Database Collaboration exchange new and updated data on a daily basis to achieve optimal synchronisation. http://www.ebi.ac.uk/embl/Contact/collaboration	14755292
0850	encode	ENCODE (the Encyclopedia Of DNA Elements) seeks to identify all protein-coding genes. The current ENCODE data set is derived from 1% of the human genome and has been selected for analysis in the pilot phase of the project. http://www.genome.gov/10005107	17372197
0476	ensembl	Ensembl is a joint project between the EMBL-EBI and the Wellcome Trust Sanger Institute that aims at developing a system that maintains automatic annotation of large eukaryotic genomes. http://www.ebi.ac.uk/ensembl	15078858
0477	entrez gene/locuslink	LocusLink provides a single query interface to curated sequence and descriptive information about genetic loci. http://www.ncbi.nlm.nih.gov/LocusLink/	14755292
0478	flybase	FlyBase is a comprehensive database for information on the genetics and molecular biology of Drosophila. http://fbserver.gen.cam.ac.uk:7081/	14755292
0249	huge	A Database of Human Unidentified Gene-Encoded Large Proteins Analyzed by Kazusa Human cDNA Project. http://www.kazusa.or.jp/huge/	14755292
		IPI provides a top level guide to the main databases that describe the proteomes of higher eukaryotic organisms. IPI effectively maintains a database of cross [illegible]	

VI. pISTil PROGRAM FLOW

This section is an overview of the **pISTil** program flow and of the most important associated messages.

1. Reads the pISTil configuration file (config_analyse.pm)

- Checks dbname, dbhost, dbuser, dbpass, path_to_databank_pattern, path_to_databank_blastX

  if success: MESSAGE: None
  RESULT: **pISTil** continues

  if failure: MESSAGE: Error in the configuration file: Check the value of the X parameter (X for the empty parameter)!
  RESULT: **pISTil** exits immediately

2. Reads the Phred parameter given in the configuration file

- Checks if it is valid

  if success: MESSAGE: **Phred** will be executed with or without base calling
  RESULT: **pISTil** continues

  if failure: MESSAGE: Error in the config file. Check the value for the **Phred** parameter!
  RESULT: **pISTil** exits immediately

3. Archive file type

- Checks if the first argument when you start **pISTil** is a zip archive and placed in the pISTil/dataset directory

  if success: MESSAGE: None
  RESULT: **pISTil** continues

  if failure: MESSAGE: Error: The zip format is not correct for $zip file.
  or Error: dataset\$zip does not exist.
  RESULT: **pISTil** exits immediately

4. Checks the bait parameter file format

- Checks if the "define_bait" or the second argument file is properly formatted

  if success: MESSAGE: None
  RESULT: **pISTil** continues

  if failure: MESSAGE: ERROR: the bait parameter file $file is not properly formatted
  RESULT: **pISTil** exits immediately

5. Defines the project and the library identifiers

- **pISTil** asks information about the project and the library to use.

  _About the project:

  - **pISTil** shows all projects in the **pISTil** database. To continue, you must choose a project identifier or create a new one.

    if success: MESSAGE: None
    RESULT: **pISTil** continues

    if failure: MESSAGE: Project id 'x' is not in the database.
    RESULT: **pISTil** exits immediately

  _About the library:

- **pISTil** shows all libraries in the **pISTil** database. To continue, you must choose a library identifier.

if success: MESSAGE: None
RESULT: **pISTil** continues
if failure: MESSAGE: Library id 'x' is not in the database.
RESULT: **pISTil** exits immediately

6. Unzips the zip archive

- **pISTil** unzips the zip archive in a tmp directory.

if success: MESSAGE: None
RESULT: **pISTil** continues
if failure: MESSAGE: Invalid Zip file:$zip
RESULT: **pISTil** exits immediately

7. Checks all raw file names according to the regex and parameters locations to define baits

- **pISTil** tries to identify the plate name, the wells locations with regex set in the configuration file.

if success: MESSAGE: None
RESULT: **pISTil** continues
if failure: MESSAGE: Regex for (plate or location) is not good, please check the configuration
RESULT: **pISTil** exits immediately

8. Checks if the plate has already been analysed by pISTil

- It's possible to analyse more than one time the same plate but with an other database for **BLAST** or an other **Phred** parameter

-**pISTil** checks if it's a new plate:
if success: MESSAGE: It's a new plate for this project
RESULT: **pISTil** continues
else, **pISTil** check three possibilities:
- the plate is analysed with an other **Phred** processing
- the plate is analysed with an other database
- the plate is analysed with an other database and an other **Phred** processing

so: if this plate is already analysed with this database:
- **pISTil** checks if it's analysed with the same **Phred** parameter
if success: MESSAGE: It's the same **Phred** analysis, **pISTil** exits
RESULT: **pISTil** exits
else: MESSAGE: This plate will be analysed with the new **Phred** parameter with the $dbname database.
RESULT: **pISTil** continues and checks if this **Phred** processing is already done.
if success: MESSAGE: No **Phred** extraction, already done with an other db.
RESULT: **pISTil** continues without **Phred** extraction
if failure: MESSAGE: **Phred** extraction
RESULT: **pISTil** continues with new **Phred** extraction
or: if this plate is analysed with a new database:
- **pISTil** checks if we have already used the same **Phred** parameter.

if success: MESSAGE: No **Phred** extraction, already done with an other db.
RESULT: **pISTil** continues without **Phred** extraction
if failure: MESSAGE: **Phred** extraction
RESULT: **pISTil** continues with new **Phred** extraction

9. Checks if the external programs are correctly installed:

- **Phred**, **Pregap4**, **extract_seq**, **blastall**
  if success: MESSAGE: None
  RESULT: **pISTil** continues
  if failure: MESSAGE: Executable $ not found!
  RESULT: **pISTil** exits immediately

10. Runs Phred

- **pISTil** runs **Phred** like : *phred -nocall -id trace_dir -cd nocall_dir*
  With: '-nocall' to disable basecalling
  '-id' to read input files from <dirname>
  '-cd' to write all scf files to <dirname>
- or **pISTil** runs **Phred like** : *phred –trim_cutoff $ -trim_alt '' –trim_fasta –sa pregapdir/pregap_full_trim_$ -id trace_dir -cd nocall_dir*
  With: '-nocall' to disable basecalling,
  '-id' to read input files from <dirname>
  '-cd' to write all scf files to <dirname>
  '-trim_cutoff $' to set trimming error probability for the 'trim_alt' option. $ can be 0.05, 0.01 etc…
  '-trim_alt' to perform sequence trimming on the current sequence.
  '-trim_fasta' to trim sequences written to sequence and quality value FASTA file called pregap_full_trim_$ with $=0.05, 0.01 ….

11. Runs Pregap4

- **pISTil** runs **Pregap4** like: *pregap4 -nowin -config $pregap_config -out_dir $pregap_dir $nocall_dir/**
  With: '-nowin' to run **Pregap4** as a batch job
  '-config' to specify the configuration file to **Pregap4**.

12. Creates all sql files to implement results data in the pISTil database

if success: MESSAGE: None
RESULT: **pISTil** continues
if failure: MESSAGE: Error: Can't create sql file!
RESULT: **pISTil** exits immediately

13. Parses all experiment files

if success: MESSAGE: None
RESULT: **pISTil** makes sql_quality, sql_trace and continues

14. Identify IST

if success: MESSAGE: None
RESULT: **pISTil** identifies preys and creates latest sql files
if failure: MESSAGE: No hit for raw file name
RESULT: **pISTil** continues with next raw files

15. pISTil finished

if success: MESSAGE: End **pISTil** analyzed at $date
RESULTS: **pISTil** asks you to add data on the database
pISTil stored data in pISTil/www/data/$idproject/
with $idproject as the project identifier choose
before the analysis.

if failure: MESSAGE: None
RESULT: **pISTil** exits

VII. pISTil FILES:

- The **pISTil** root directory contains the following files and folders:

README	- **pISTil** README
config_analyse.pm	- The **pISTil** configuration file
define_bait	- The bait configuration file
ist_analyse.pl	- The **pISTil** executable
pregap4_pistil.config	- The local **Pregap4** config file for **pISTil**
#DIRECTORY	
dataset/	- Contains zip archive files to analyze
dataset/HCV1.zip	- Zip archive file given as a first demo
dataset/HCV2.zip	- Zip archive file given as a second demo
dataset/README	- Dataset README
db/	- Contains scripts to build the pISTil database
db/create_database.csh	- Shell script to build the pISTil database
db/data_IST.sql	- SQL file with default data
db/database_IST.sql	- SQL file that contains the main table structure for the **pISTil** database
docs/	- Contains the **pISTil** documentation
docs/pISTil_doc.pdf	- **pISTil** documentation
localdb/	- Contains local databases for BlastX
localdb/ensembl/	- Directory for Ensembl database
localdb/camjedb/	- Directory for Camjedb database
localdb/cygd/	- Directory for CYGD database
localdb/ddbj-embl-genbank/	- Directory for DDBJ EMBL GenBank database
localdb/encode/	- Directory for ENCODE database
localdb/entrez_gene_locuslink/	- Directory for LocusLink database
localdb/flybase/	- Directory for FlyBase database
localdb/huge/	- Directory for huge database
localdb/international_protein_index/	- Directory for IPI database
localdb/mgd-mgi/	- Directory for MGI database
localdb/omim/	- Directory for OMIM database
localdb/other/	- Directory for other database
localdb/refseq/	- Directory for RefSeq database
localdb/rfam/	- Directory for Rfam database
localdb/rgd/	- Directory for RGD database

localdb/sgd/ - Directory for SGD database
localdb/uniparc/ - Directory for Uniprot Archive database
localdb/uniprot_knowledge_base/ - Directory for Uniprot database
localdb/wormbase/ - Directory for WormBase database
localdb/pattern/ - Directory for pattern database (**BlastN**)

/www - Contains all files for the **pISTil** web interface
/www/data - Contains all results after the **pISTil** analysis which are displayed in the web interface.
/www/img - Contains images for the interface.
/www/inc - Contains all PHP include files for the interface.
/www/traceviewer - Contains all scripts for the **BMC TraceViewer**.

- Before running the first analysis with the dataset given in example, you generate:

localdb/refseq/refseq_human_prot.phr |
localdb/ refseq /refseq_human_prot.pin |
localdb/ refseq /refseq_human_prot.pnd |
localdb/ refseq /refseq_human_prot.pni |- refseq database for **BlastX**
localdb/ refseq /refseq_human_prot.psd |
localdb/ refseq /refseq_human_prot.psi |
localdb/ refseq /refseq_human_prot.psq |
localdb/pattern/pPC86.nhr |
localdb/pattern/pPC86.nin |
localdb/pattern/pPC86.nsd |- Pattern database for **BlastN**
localdb/pattern/pPC86.nsi |
localdb/pattern/pPC86.nsq |
localdb/formatdb.log - **formatdb** logfile (optional)

- After the first run with the HCV dataset, you will generate:

tmp - Contains temporary files
www/data/1/ - '1' corresponds to the project identifier in the **pISTil** database
www/data/1/outfile_blast_dir/ - Contains all **Blast** result files (depends on your **pISTil** configuration)
www/data/1/phred_scf_dir/ - Contains all scf trace files
www/data/1/qual_dir/ - Contains all html quality files
www/data/1/sql_dir/ - Contains all sql files
www/data/1/pISTil.log - **pISTil** log (depends on your **pISTil** configuration)

- After a second run on the same project, you have more:

www/data/1/sql_dir_X - Contains all sql files created in X date time.

VIII. BUGS AND PROBLEMS

Some crash can occur when you run **pISTil**. Errors may be due to incorrectly configured programs required.

1. Environment variable STADLIB

If the run stops prematurely, displaying the message:

```
Error: Environment variable STADLIB not set. Died at ist_analyse.pl line 226
```

Then you need to define the 'STADLIB' environment variable. Please follow instructions in II.8.

2. Stash not found

If the run stops prematurely with the message :

```
usr/local/bin/pregap4: line 123: exec: stash: not found
main::extract_pregap4
main::get_Extracts
```

Then you must be sure that you have define in your environment variable:
LD_LIBRARY_PATH, TCL_LIBRARY, TK_LIBRARY and
$STADENROOT/staden.profil (please see **Staden** Instructions for more details).

Please report **pISTil** problems and bugs to johann.pellet@inserm.fr

Annex

Annex 1: pISTil Entity-relationship (ER) diagram

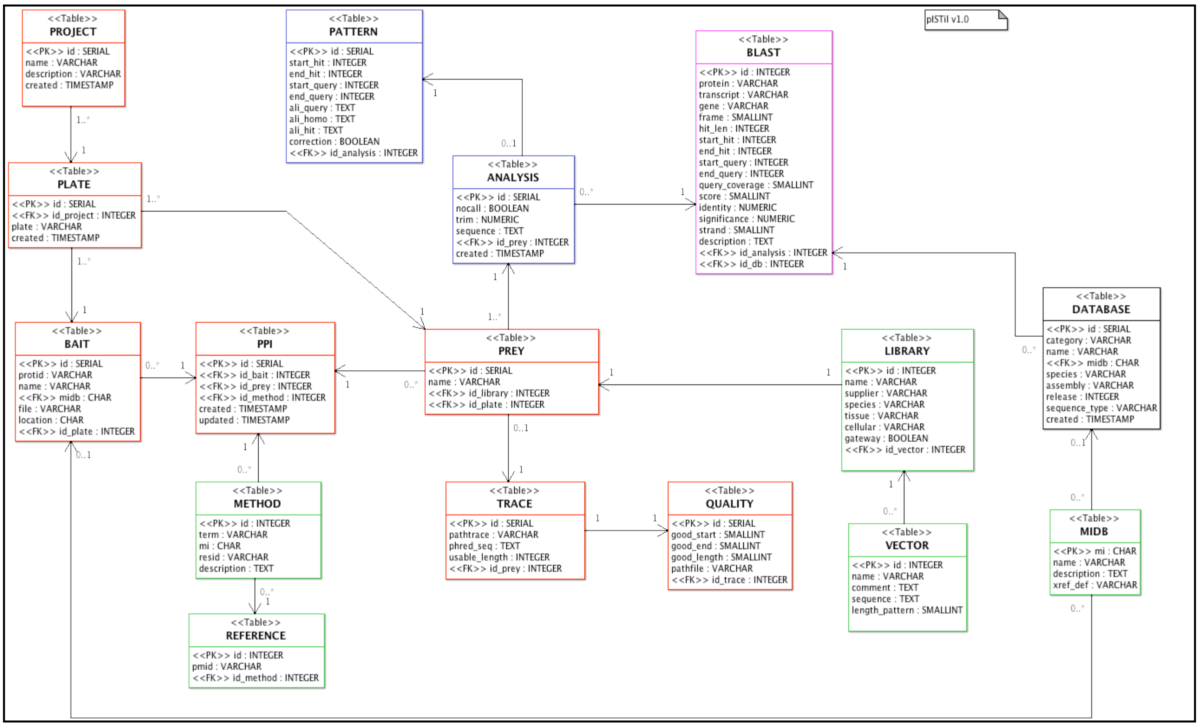

Short table description**:**

analysis: Stores different sequence analysis for prey.
One prey can be analysed more than one time with different databases or **Phred** extractions.

bait: Stores bait information and location in its plate.
By reference, the bait protein corresponds to the investigator protein of interest and is fused to the DNA Binding Domain (BD) of the transcription factor Gal4 (Gal4-BD). It is assayed against a cDNA library encoding proteins fused to the Activation Domain (AD) of the transcription factor Gal4 (Gal4-AD), that are referenced as prey-proteins. To identify the bait (protein identifier and name), **pISTil** uses the 'define_bait' file.
Each bait can interact with one and only one prey during an analysis.

blast: Stores BLAST results.
Each analysed sequence is aligned with **BLASTX** (only in the three positive frames) against a protein database to identify an IST.

database: Stores database information.
Preys are identified using **BLASTX** against a protein database.

library: Stores libraries information.
A library consists of a collection of protein-encoding sequences that represent all the proteins expressed in a particular organism, tissue and/or cellular type.

method: Stores method information.
All information in this table come from PSI-MI 2.5 methods information.
midb: Stores PSI-MI database information.

Database collecting nucleic or amino acid sequence mainly derived from genomic sequence.

pattern: Stores pattern information.
During the trace analysis the first step consists of looking for a sequence corresponding to the last nucleotids of Gal4-AD in the trace sequence (by **BLASTN** alignment), which is defined as the pattern.

plate: Stores information about plates.
A traditional two-hybrid plate contains 96 wells, so one plate can contain between 1 and 96 bait(s).

ppi: Stores physical interaction between the bait and the prey.
It corresponds to a protein-protein interaction (ppi) between a given bait protein (fused to Gal4-BD) and a prey protein (fused to Gal4-AD). If bait and prey interact, the two functional domains of Gal4 are brought closer, leading to the expression of a reporter gene in the yeast two hybrid system.

prey: Stores prey information.
The prey protein is fused to the activation domain (AD) of the transcription factor (Gal4-AD). It can either be a known protein in the case of a yeast two-hybrid assay, in order to test by a priori the interaction between two known proteins. It can also be an unknown protein, encoded by a cDNA of a yeast two-hybrid library.

project: Stores generic project information.
A project includes the analysis of one or several plate(s).

quality: Stores quality information sequence.
Each trace is analysed to define the sequence quality.

reference: Stores the bibliographic reference of a method.

trace: Stores trace information.

pISTil tries to identify each prey thanks to traces. These traces come from the sequencing of the cDNA encoding the prey protein fused to Gal4-AD (obtained by a PCR on positive yeast colonies of the yeast two-hybrid screen). **pISTil** uses **Extract_seq** (**Pregap4** module) to extract the sequence component from traces and experiment files.

vector: Stores vectors information.
cDNA libraries are cloned into a yeast two-hybrid vector, allowing the expression of a prey protein fused to Gal4-AD. The resulting vectors, thus composed of a library vectors, are transformed in yeast in order to be screened by the two-hybrid method.

Annex 2: PostgreSQL table listing

pISTil uses 16 tables to store and to process all the data. Below is a listing of each table.

Table	Field	Type	Extra	Description
analysis				
	id	Serial	Primary Key	Unique analysis identifier
	nocall	Boolean		Phred extraction without base calling
	trim	Numeric		Phred trim cutoff
	sequence	Text		Phred sequence extraction
	id_prey	Integer	Foreign key	Refers to Prey identifier
	created	Timestamp		Creation date
bait				
	id	Serial	Primary key	Unique bait identifier
	protid	Varchar(100)		Protein identifier from public database
	name	Varchar(100)		Bait name (according to the 'define_bait' file)
	midb	Char(4)	Foreign key	PSI-MI identifier for public database
	file	Varchar(150)		Raw file name
	location	Char(3)		Location within the plate
	id_plate	Integer	Foreign key	Refers to Plate identifier
blast				
	id	Integer	Primary key	Unique blast identifier
	protein	Varchar(15)		Protein ID blast hit
	transcript	Varchar(15)		Transcript ID blast hit (if exists)
	gene	Varchar(15)		Gene ID blast hit (if exists)
	frame	Smallint		Frame (0,1 or 2)
	hit_len	Integer		Length of the hit
	start_hit	Integer		Start hit location
	end_hit	Integer		End hit location
	start_query	Integer		Start query location
	end_query	Integer		End query location
	query_coverage	Smallint		Coverage
	score	Smallint		Blast score
	identity	Numeric		Blast identity
	significance	Numeric		Blast expectation value
	strand	Smallint		Blast strand
	description	Text		Protein description (if exists)
	id_analysis	Integer	Foreign key	Refers to Analysis identifier
	id_db	Integer	Foreign key	Refers to Database identifier
database				
	id	Serial	Primary key	Unique database identifier
	category	Varchar(100)		Database midb name
	name	Varchar(255)		Unique database name
	midb	Char(4)	Foreign key	PSI-MI identifier for public database
	species	Varchar(100)		Name of the species
	assembly	Varchar(100)		Assembly build name
	release	Integer		Release number
	sequence_type	Varchar(80)		Sequence type (peptidic or nucleotidic)

	created	Timestamp		Creation date
library				
	id	Integer	Primary key	Unique library identifier
	name	Varchar(150)		Library's name
	supplier	Varchar(150)		Supplier if you bought it
	species	Varchar(100)		Species
	tissue	Varchar(150)		Tissue
	cellular	Varchar(150)		Cellular
	gateway	Boolean		If the library is gateway compatible
	id_vector	Integer	Foreign key	Refers to vector identifier
method				
	id	Integer	Primary key	Unique method identifier
	term	Varchar(100)		Name (according to PSI-MI)
	mi	Char(4)		PSI-MI identifier
	resid	Varchar		Reference definition
	description	Text		Description
midb				
	mi	Char(4)		PSI-MI database identifier
	name	Varchar(40)		Name (according to PSI-MI)
	description	Text		Description
	xref_def	Varchar(15)		PMID (PubMed Identifier).
pattern				
	id	Serial	Primary key	Unique pattern identifier
	start_hit	Integer		Start hit location
	end_hit	Integer		End hit location
	start_query	Integer		Start query location
	end_query	Integer		End query location
	ali_query	Text		Nucleic query sequence aligned
	ali_homo	Text		Homology sequence
	ali_hit	Text		Nucleic hit sequence aligned
	correction	Boolean		pISTil correction to find cDNA start
	id_analysis	Integer	Foreign key	Refers to analysis identifier
plate				
	id	Serial	Primary key	Unique plate identifier
	id_project	Integer	Foreign key	Refers to Project identifier
	plate	Varchar(15)		Plate containing baits
	created	Timestamp		Creation date in the database
ppi				
	id	Serial	Primary key	Unique ppi identifier
	id_bait	Integer	Foreign key	Refers to Bait identifier
	id_prey	Integer	Foreign key	Refers to Prey identifier
	id_method	Integer	Foreign key	Refers to Method identifier
	created	Timestamp		Creation date in the database
	updated	Timestamp		Last Update date
prey				
	id	Serial	Primary key	Unique prey identifier
	name	Varchar(100)		Raw file name
	id_library	Integer	Foreign key	Refers to Library identifier
	id_plate	Integer	Foreign key	Refers to Plate identifier

project				
	id	Serial	Primary Key	Unique project identifier
	name	Varchar(100)		Name of the project
	description	Varchar(255)		Description of the project
	created	Timestamp		Creation date
quality				
	id	Serial	Primary key	Unique quality identifier
	good_start	Smallint		Start location of the good quality sequence
	good_end	Smallint		End location of the good quality sequence
	good_length	Smallint		Length of the good quality sequence
	pathfile	Varchar(150)		Path to the quality html file
	id_trace	Integer	Foreign key	Refers to Trace identifier
reference				
	id	Integer	Primary key	Unique pub_ref identifier
	pmid	Varchar(15)		PMID paper reference
	id_method	Integer	Foreign key	Refers to method identifier
trace				
	id	Serial	Primary key	Unique trace identifier
	pathtrace	Varchar(150)		Path to the raw file
	extract_seq	Text		Nucleic sequence extracted by Pregap4
	usable_length	Integer		Length of good quality sequence
	id_prey	Integer	Foreign key	Refers to Prey identifier
vector				
	id	Integer	Primary key	Unique vector identifier
	name	Varchar(80)		Vector name
	comment	Text		Some comments
	sequence	Text		Nucleic sequence before cDNA
	length_pattern	Smallint		Length of pattern

Annex 3: Config_analyse.pm example, working with both HCV datasets

- **dbname**: 'pistil'
- **dbhost**: 'localhost'
- **dbuser**: 'IST_user'
- **dbpass**: 'istdb'
- **temp_dir**: 'tmp/
- **path_to_pregap_config**: 'pregap4_pistil.config'
- **path_to_databank_pattern**: 'localdb/pattern'
- **path_to_databank_blastX**: 'localdb/refseq/refseq_human_prot'
- **MI_method**: '0018'
- **phred_arg**: '-nocall'
- **dataset_dir**: 'dataset/'
- **regex_plate**: '^(\w+)\-\w\d\d\-.*'
- **regex_location**: '^\w+\-(\w\d\d)\-.*'
- **save_BLASTN**: 'n'
- **save_BLASTX**: 'n'
- **log_file**: 'y'

<u>Annex 4</u>: Define_bait examples

Configuration for the plate HCV15_1_96 from HCV.zip file.

First well	Last well	Bait product	Bait proteinid	PSIMI database id
A01	H12	NS3	CAB46677	0475

Configuration for the plate MARIE1 from HCV2.zip file.

First well	Last well	Bait product	Bait proteinid	PSIMI database id
A01	F12	NS3	CAB46677	0475
G01	H02	NS2	CAB46677	0475
H03	H12	NS3	CAB46677	0475

If you create a new zip file with all traces from HCV1.zip and HCV2.zip you have to configure the define_bait like this:

First well	Last well	Bait product	Bait proteinid	PSIMI database id
--HCV15_1_96				
A01	H12	NS3	CAB46677	0475
--MARIE1				
A01	F12	NS3	CAB46677	0475
G01	H02	NS2	CAB46677	0475
H03	H12	NS3	CAB46677	0475

<u>Annex 5:</u> config.inc example, working with both HCV datasets

$HOST_NAME = "localhost";
$DATABASE_NAME = "pistil";
$DATABASE_USER = "IST_user";
$DATABASE_PASSWORD = "istdb";

//Paths for MAMP user
$LOCAL_DIR = "/Applications/MAMP/htdocs/pISTil/www/";
$FORMATDB_EXEC = "/usr/local/bin/formatdb";
$LOCALDB_PATH = "/Applications/MAMP/htdocs/pISTil/localdb/";

Annex 6: **PSI-MI from Ontology browser for sequence databases**

List of databases commonly used to cross reference interaction data. For more information: http://www.ebi.ac.uk/ontology-lookup/browse.do?ontName=MI

MI	DATABASE NAME	Link
**0249**	huge	http://www.kazusa.or.jp/huge/
**0464**	cygd	http://mips.gsf.de/proj/yeast/CYGD
**0475**	ddbj/embl/genbank	http://www.ebi.ac.uk/embl/Contact/collaboration
**0476**	ensembl	http://www.ebi.ac.uk/ensembl
**0477**	entrez gene/locuslink	http://www.ncbi.nlm.nih.gov/LocusLink/
**0478**	flybase	http://fbserver.gen.cam.ac.uk:7081/
**0479**	mgd/mgi	http://www.informatics.jax.org/
**0480**	omim	http://www.ncbi.nlm.nih.gov/entrez/query.fcgi?db=OMIM
**0481**	refseq	http://www.ncbi.nlm.nih.gov/RefSeq/
**0482**	rfam	http://www.sanger.ac.uk/Software/Rfam/
**0483**	rgd	http://rgd.mcw.edu/
**0484**	sgd	http://www.yeastgenome.org/
**0485**	uniparc	http://www.ebi.ac.uk/uniparc/
**0486**	uniprot knowledge base	http://www.expasy.uniprot.org/
**0487**	wormbase	http://www.wormbase.org/
**0675**	IPI	http://www.ebi.ac.uk/IPI/IPIhelp.html
**0850**	encode	http://www.genome.gov/10005107
**0924**	camjedb	http://www.sanger.ac.uk/Projects/C_jejuni/

<u>Annex 7:</u> Vector pPC86 used for the HCV examples and its pattern

We selected the 87 last nucleotides of *GAL4-AD* encoding-sequence as the pattern to determine the Gal4-AD frame. This sequence was chosen as it is downstream of the Y2HAD primer used for PCR and sequencing the cDNA cloned in pPC86.

The sequence next the pattern was analysed by **BlastX** against a protein database corresponding to the organism of library screened (in our case, the human).

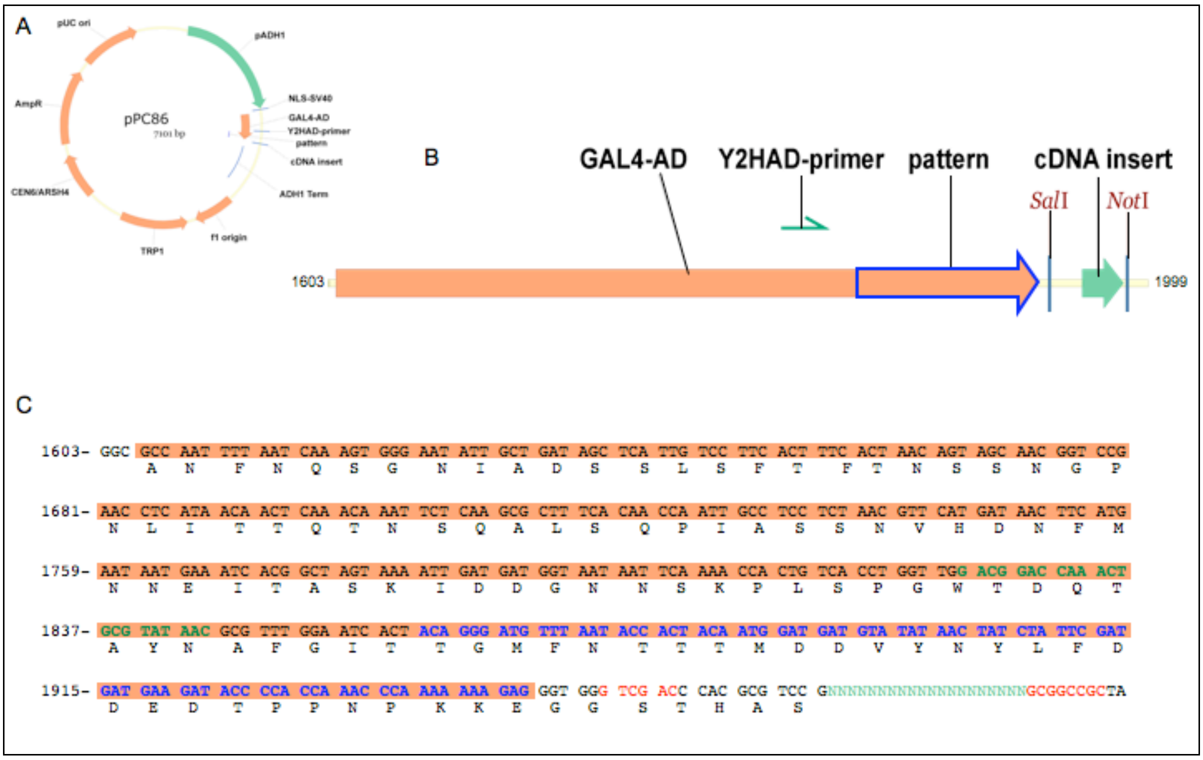

A. pPC86 vector map

B. Zoom of pPC86 cloning site (nt 1603-1999). Sequences of *GAL4-AD* and cDNA restriction sites are labeled. Also shown, the Y2HAD primer binding site (green) and the pattern (blue).

C. Nucleotidic (and amino acid) sequence of pPC86 cloning site. Sequence highlighted in orange corresponds to *GAL4-AD* (aa 768-881 of Gal4). Sequences in green represents Y2HAD primer binding site, in blue the pattern and in red the restriction sites of cDNA in pPC86.

<u>Annex 8:</u> pISTil workflow schema

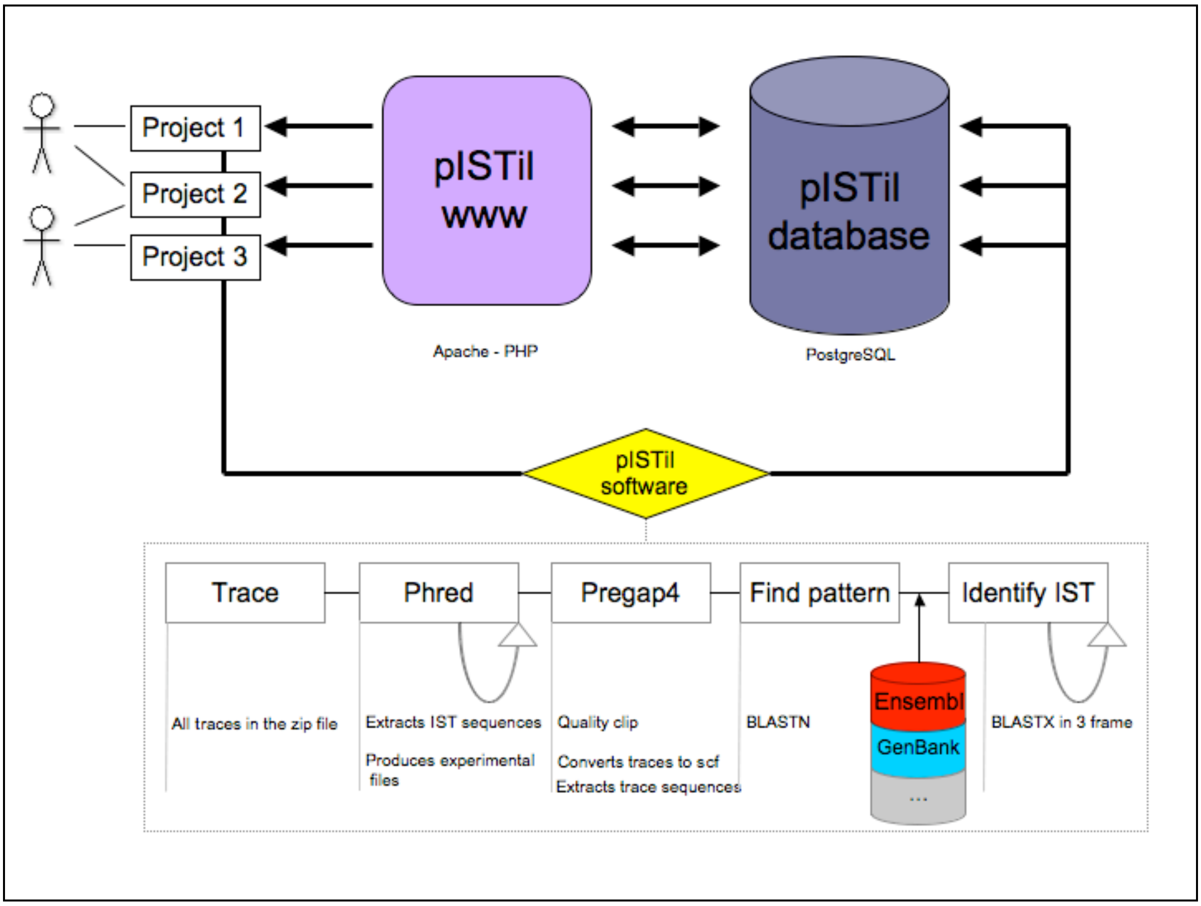

pISTil is organized around three major components. The **pISTil** software analyses chromatogram files (traces) organized by project. In this case, three different projects are shown with two users. The **pISTil** web application can provide visualization for any number of projects. The **pISTil** database shares all analysis information. We represent by an arrow the possibility to use **pISTil** more than one time with the same dataset changing **Phred** base calling argument or database for the **BLASTX**.

<u>Annex 9:</u> The trace sequence, according to several Phred processing options

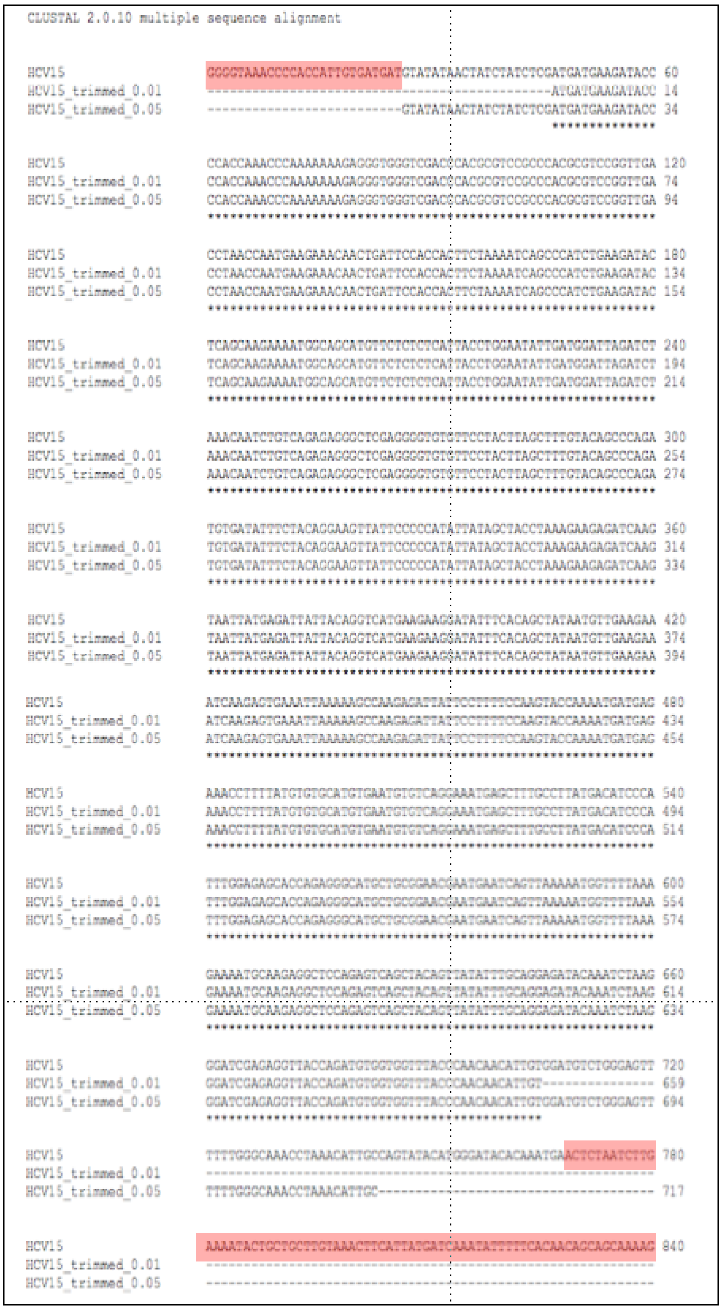

pISTil can analyse traces with different parameters, like **Phred** trimming. Here you can visualize a **ClustalW** alignment that highlights the difference for the same sequence between different extractions. HCV15 is extracted without trimming, HCV15_trimmed_0.01 is extracted with trim cutoff 0.01 and HCV15_trimmed_0.05 is extracted with trim cutoff 0.05. **Phred** finds the longest fragment in a sequence where the estimated error rate is below the cutoff value. For more explanation about the **Phred** trimming, please see the **Phred** documentation.

<u>Annex 10:</u> Interaction Sequence Tag (IST) identification pipeline statistics

The number of ISTs, distinct protein-protein interactions (ppi) and distinct host proteins generated by Infection MAPping (I-MAP) team, with the complete HCV dataset, are given before filtering, for each filter and with all filters (significative ppi).

**IST identification by pISTil**			
	ISTs	ppi	proteins
Without filtering	1158	477	395
Identity>=80%, e-value<=1E-10	578	208	186
In frame	653	243	207
**All filters (significative)**	443	132	117

<u>Annex 11:</u> Schema of the BLASTN and the split correction to find the cDNA start.

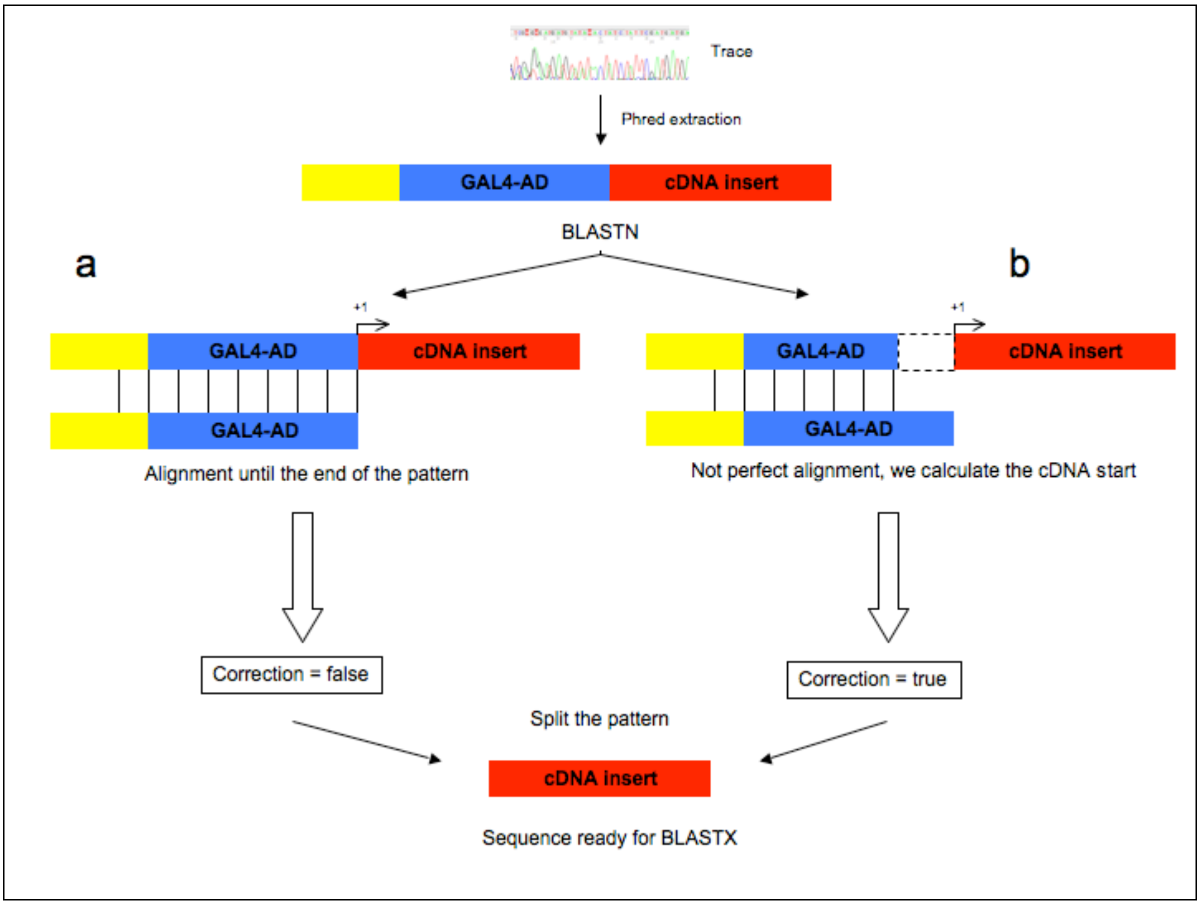

We use **BLASTN** to search the pattern GAL4-AD placed before the cDNA. If the alignment is perfect, the term correction is set to false (a). However, since the pattern is at the beginning of the sequence, it is possible that the alignment is not perfect (b). If the end of the alignment between the pattern and the IST is not the end of the pattern, we calculate the cDNA start by adding the number of missing bases, and the term correction is set to true.
